# From cytogenetics to cytogenomics: whole-genome sequencing as a first-line test comprehensively captures the diverse spectrum of disease-causing genetic variation underlying intellectual disability

**DOI:** 10.1186/s13073-019-0675-1

**Published:** 2019-11-07

**Authors:** Anna Lindstrand, Jesper Eisfeldt, Maria Pettersson, Claudia M. B. Carvalho, Malin Kvarnung, Giedre Grigelioniene, Britt-Marie Anderlid, Olof Bjerin, Peter Gustavsson, Anna Hammarsjö, Patrik Georgii-Hemming, Erik Iwarsson, Maria Johansson-Soller, Kristina Lagerstedt-Robinson, Agne Lieden, Måns Magnusson, Marcel Martin, Helena Malmgren, Magnus Nordenskjöld, Ameli Norling, Ellika Sahlin, Henrik Stranneheim, Emma Tham, Josephine Wincent, Sofia Ygberg, Anna Wedell, Valtteri Wirta, Ann Nordgren, Johanna Lundin, Daniel Nilsson

**Affiliations:** 1 Department of Clinical Genetics, Karolinska University Hospital, Stockholm, Sweden; 2 Department of Molecular Medicine and Surgery, Karolinska Institutet, Stockholm, Sweden; 3 Center for Molecular Medicine, Karolinska Institutet, Stockholm, Sweden; 4 Science for Life Laboratory, Karolinska Institutet, Stockholm, Sweden; 5 Department of Molecular and Human Genetics, Baylor College of Medicine, Houston, Texas USA; 6 The Department of Women’s and Children’s Health, Karolinska Institutet, Stockholm, Sweden; 7 Centre for Inherited Metabolic Diseases, Karolinska University Hospital, Stockholm, Sweden; 8 Department of Biochemistry and Biophysics, National Bioinformatics Infrastructure Sweden, Science for Life Laboratory, Stockholm University, Stockholm, Sweden; 9 Science for Life Laboratory, School of Engineering Sciences in Chemistry, Biotechnology and Health, KTH Royal Institute of Technology, Stockholm, Sweden; 10 Science for Life Laboratory, Department of Microbiology, Tumor and Cell biology, Karolinska Institutet, Stockholm, Sweden

**Keywords:** Whole-genome sequencing, Intellectual disability, Monogenic disease, Copy number variation, Structural variation, Single nucleotide variant, Uniparental disomy, Repeat expansion

## Abstract

**Background:**

Since different types of genetic variants, from single nucleotide variants (SNVs) to large chromosomal rearrangements, underlie intellectual disability, we evaluated the use of whole-genome sequencing (WGS) rather than chromosomal microarray analysis (CMA) as a first-line genetic diagnostic test.

**Methods:**

We analyzed three cohorts with short-read WGS: (i) a retrospective cohort with validated copy number variants (CNVs) (cohort 1, *n* = 68), (ii) individuals referred for monogenic multi-gene panels (cohort 2, *n* = 156), and (iii) 100 prospective, consecutive cases referred to our center for CMA (cohort 3). Bioinformatic tools developed include FindSV, SVDB, Rhocall, Rhoviz, and vcf2cytosure.

**Results:**

First, we validated our structural variant (SV)-calling pipeline on cohort 1, consisting of three trisomies and 79 deletions and duplications with a median size of 850 kb (min 500 bp, max 155 Mb). All variants were detected. Second, we utilized the same pipeline in cohort 2 and analyzed with monogenic WGS panels, increasing the diagnostic yield to 8%. Next, cohort 3 was analyzed by both CMA and WGS. The WGS data was processed for large (> 10 kb) SVs genome-wide and for exonic SVs and SNVs in a panel of 887 genes linked to intellectual disability as well as genes matched to patient-specific Human Phenotype Ontology (HPO) phenotypes. This yielded a total of 25 pathogenic variants (SNVs or SVs), of which 12 were detected by CMA as well. We also applied short tandem repeat (STR) expansion detection and discovered one pathologic expansion in *ATXN7*. Finally, a case of Prader-Willi syndrome with uniparental disomy (UPD) was validated in the WGS data.

Important positional information was obtained in all cohorts. Remarkably, 7% of the analyzed cases harbored complex structural variants, as exemplified by a ring chromosome and two duplications found to be an insertional translocation and part of a cryptic unbalanced translocation, respectively.

**Conclusion:**

The overall diagnostic rate of 27% was more than doubled compared to clinical microarray (12%). Using WGS, we detected a wide range of SVs with high accuracy. Since the WGS data also allowed for analysis of SNVs, UPD, and STRs, it represents a powerful comprehensive genetic test in a clinical diagnostic laboratory setting.

## Background

Up to 80% of rare diseases have a genetic background [1], and in the last decade, our understanding of the underlying genetic lesions has increased rapidly. Since human genetic variation is abundant and diverse, ranging from small variants affecting only one or a few base pairs, i.e., single nucleotide variants (SNVs), small insertions and deletions (INDELs) to large structural variants (SVs) affecting thousands or millions of nucleotides [2–4], diagnostic tests need to capture the full spectrum of variation. Technological advancements in high-throughput sequencing (massively parallel sequencing, MPS) has allowed for comprehensive sequencing of many individuals from various populations [5–7] highlighting the vast complexity and abundance of rare and common genetic variation [8, 9].

In genetic diagnostics, the current toolbox includes a great variety of cytogenetic and molecular methodologies. Chromosomal microarray analysis (CMA), either comparative genomic hybridization (CGH) or SNP arrays, has long been the first-tier test used to identify copy number variants (CNVs) in individuals with intellectual disability and neurodevelopmental disorders [10–12]. In recent years, MPS-based assays have been increasingly used in rare disease diagnostics, because of their high throughput and cost effectiveness in screening multi-gene panels for hereditary disorders [13]. Whole-exome sequencing (WES) is more and more widely used in clinical labs as a first-tier test, allowing detection of SNVs, INDELs, and CNVs covering multiple exons (typically > 2) [14–16]. In a recent meta-analysis, the diagnostic yield of WES in 3350 individuals with neurodevelopmental disorders was 36%, ranging from 8 to 90%, with the higher yield obtained after trio analysis of the affected individuals and both parents [17]. Even though isolated WES or WES in combination with CMA enables effective detection of both SNVs and CNVs [18], some types of variants such as balanced chromosomal rearrangements, small CNVs (< 2 exons), and trinucleotide repeat expansion disorders will be missed. Depending on the specific clinical presentation and the preferences of the referring physician and the laboratory performing the test, other assays such as G-banded karyotyping, fluorescence *in situ* hybridization (FISH), *FMR1* CGG repeat expansion analysis, PCR-based single gene analysis, and whole-genome sequencing (WGS) may then be performed [19].

Each individual method has intrinsic specific limitations which may result in causal variants being missed (e.g., mosaicism in probands) or misinterpreted (e.g., gene copy number gains consistent with triplications or higher order gains can be challenging to distinguish from duplications [20]), resulting in sub-optimal clinical management and imprecise genetic counseling [21]. In addition, the possibility of dual diagnosis due to multi-locus variation [22] has been reported for up to 5% of individuals with Mendelian diseases and can explain apparent phenotypic expansion [23]. In research, WGS has been used to detect a wide range of mutations, including copy number variations [24–26] as well as balanced chromosomal rearrangements such as translocations [27, 28], inversions [29], and short tandem repeats (STRs) [30]. A few studies have performed CNV calling from WGS in small cohorts, showing diagnostic rates of 15% (10/79) [24], 33% (20/60) [31], and 14% (7/50) [32]. Although WGS is the most comprehensive test currently available for molecular diagnostics in clinical practice, the routine use of WGS largely remains limited to SNVs and INDELs [33, 34]. This is because WGS-based SV detection in a clinical setting remains challenging, partly because of the low precision and sensitivity of the SV callers and lack of normal variant databases, but also due to the limited standardization and benchmarking of the various pipelines [35].

In this study, we investigate the application of WGS as a first-line test in intellectual disability and compare the outcome with results from CMA. In aggregate, the results highlight the ability to capture a broad range of genetic variation including both large and small CNVs, SNVs, balanced rearrangements, repeat expansions, and uniparental disomy (UPD). In a prospective unselected cohort of 100 patients referred to our laboratory for CMA, the overall diagnostic yield of WGS was 27% compared to 12% obtained with our standard clinical CMA.

## Methods

### Study subjects

Clinical Genetics (Karolinska University Hospital, Stockholm, Sweden) is a tertiary center where genome-wide screening for CNVs by CMA is used as a first-line test for individuals with suspected rare genetic disease, neurodevelopmental disorders (NDD), and malformation syndromes. For individuals with a high suspicion of a monogenic disease, WGS (with *in silico* gene panel analysis) is performed as the first-line test. Overall, roughly 1000 CMAs and 500 WGS analyses are performed annually. In this study, all included patients were initially referred for clinical diagnostic testing and, when possible, parental analysis was performed to assess the parental origin of identified variants. Three cohorts were investigated:

- Cohort 1, “The validation cohort”, consisted of 68 individuals harboring three trisomies and 79 CNVs previously detected by CMA or multiplex ligation-dependent probe amplification (MLPA).
- Cohort 2, “The monogenic disease study cohort”, consisted of 156 individuals referred for WGS due to a clinical suspicion of monogenic disease within the areas of neuromuscular disorders, connective tissue disorders, unknown syndromes, skeletal dysplasias, hereditary cancer, or other rare suspected Mendelian conditions.
- Cohort 3, “The prospective study cohort”, consisted of the first 100 unselected individuals that were clinically referred for CMA in 2017.

The clinical parameters of the three cohorts are summarized in Table 1 and detailed information is given in Additional file 1: Table S1. The local ethical board in Stockholm, Sweden, approved the study (approval numbers KS 2012/222-31/3 and 2012/2106-31/4).

**Table 1:** Clinical parameters of included cases

Clinical	Cohort 1 (*n* = 68)	Cohort 2 (*n* = 156)	Cohort 3 (*n* = 100)
Gender (F/M)	44%/56%	44%/50%*	37%/63%
Main phenotype			
NDD	21 (31%)	2 (1%)	40 (40%)
NDD+	13 (19%)	0	38 (38%)
Syndrome	20 (29%)	53 (34%)	8 (8%)
Growth abnormality	5 (7%)	0	4 (4%)
Metabolic crisis	0	0	3 (3%)
Endocrine abnormality	2 (3%)	0	1 (1%)
Internal malformations	1 (1%)	0	2 (2%)
Neuromuscular abnormality	1 (1%)	32 (21%)	2 (2%)
CTD	0	28 (18%)	0
Hereditary cancer	3 (4%)	33 (21%)	0
Other**	2 (3%)	8 (5%)	2 (2%)

*F* female, *M* male, *NDD* neurodevelopmental disorder, *NDD+* syndromic NDD, *CTD* connective tissue disorder. *6% no gender information (fetal sample or disorder of sex development). **Epilepsy, disorder of sex development, eye disorder, immunological disorder, and skin disease

### Chromosomal microarray analysis

Genomic DNA was isolated from whole blood using standardized protocols and used for array comparative genomic hybridization (array-CGH) analysis. A 4 × 180K custom oligonucleotide microarray with whole-genome coverage and a median probe spacing of approximately 18 kb was used (AMADID:031035, Oxford Gene Technology, Begbroke, Oxfordshire, UK). This array design is used as a routine diagnostic tool at the Department of Clinical Genetics, Karolinska University Hospital, Stockholm, Sweden. For sample RD_P409, a medical exome 1 × 1 M Agilent oligonucleotide microarray was used (AMADID:068073, Oxford Gene Technology, Begbroke, Oxfordshire, UK). The medical exome 1 × 1 M array is an exon-focused array used for targeted analysis of deletions/duplications falling below the resolution of the 4 × 180K standard microarray. The probes have been selected to allow for single exon resolution in 4645 known disease-causing genes.

The control DNA used for the array-CGH experiment consisted of a mix of sex-matched DNA from several healthy individuals pooled together (Promega, Madison, WI, USA). Sample labelling (CGH labelling kit for oligo arrays, Enzo Life Sciences, Farmingdale, NY, USA), hybridization, and slide washing (Oligo aCGH/ChIP-on-Chip Wash Buffer Kit, Agilent Technologies, Wilmington, DE, USA) were performed according to the manufacturers’ recommendations. Slides were scanned using the Agilent Microarray Scanner (G2505C, Agilent technologies, USA) with 3 μm resolution. Raw data were normalized using Feature Extraction Software v10.7.3.1 (Agilent Technologies, Santa Clara, CA, USA), and log2 ratios were calculated by dividing the normalized intensity in the sample by the mean intensity across the reference sample. The log2 ratios were plotted and segmented by circular binary segmentation in the CytoSure Interpret software v4.10 (Oxford Gene Technology, Oxfordshire, UK). Oligonucleotide probe positions were annotated according to the human genome assembly hg19 [36]. For the 4×180K microarray, three consecutive aberrant probes with a log2 ratio cutoff of − 0.65 for deletions and 0.35 for duplications were called, giving a practical lower resolution of about 50 kb. The clinical relevance of all CNVs was classified into five categories; benign, likely benign, variant of uncertain significance (VUS), likely pathogenic, and pathogenic, according to the American College of Medical Genetics and Genomics (ACMG) guidelines [37] and based upon the size of aberration, gene content, inheritance, and available information in medical literature and different databases: the Database of Genomic Variants (DGV) [38], the Database of Chromosomal Imbalance and Phenotype in Humans using Ensembl Resources (DECIPHER) [39], Online Mendelian Inheritance in Man (OMIM) [40], and an in-house database with variants from ~ 8000 analyzed cases.

### Multiplex ligation-dependent probe amplification

Multiplex ligation-dependent probe amplification (MLPA) analysis was performed using the available probe set for selected assays (P090 (*BRCA2*), P256 (*FLCN*), P003 (*MLH1*), and ME028 (PWS) MRC-Holland, Amsterdam, Netherlands). MLPA was carried out according to the supplier’s recommendations, with the exception that the PCR reactions were performed in a 25-μl reaction volume. Amplification products were quantified by capillary electrophoresis on an ABI3500xL Genetic Analyzer (Applied Biosystems, Thermo Fisher Scientific, Waltham, MA, USA) with accompanying software. The tracing data was imported into and analyzed in GeneMarker software v1.7 (SoftGenetics LLC, State College, PA, USA). The normalized quotients for the different probes were considered as a deletion when below 0.75 and a duplication when above 1.3.

### Genotyping

Genomic DNA from individual RD_P432 and her mother were analyzed using 12 polymorphic microsatellite markers located on chromosome 15 (D15S1035, D15S128, D15S1513, D15S97, D15S1002, D15S165, D15S1007, D15S123, D15S1024, D15S992, D15S1028, and D15S978). Primers were pooled and amplified using Type-it Microsatellite PCR Kit according to the manufacturer’s instructions (QIAGEN, Hilden, Germany). PCR products were analyzed using 3500xL Genetic Analyzer and GeneMapper v5 according to the manufacturer’s protocol (Applied Biosystems).

### Short-read whole-genome sequencing

Genomic DNA from whole blood was sequenced using the Illumina Hiseq X Ten platform, using a 30× PCR-free paired-end WGS protocol. The patients of cohort 1 (*n* = 68) were sequenced at the National Genomics Infrastructure (NGI), Stockholm, Sweden [41], and the patients of cohort 2 (*n* = 156) and cohort 3 (*n* = 100) were sequenced at Clinical Genomics, Stockholm, Sweden [42]. The resulting WGS data was preprocessed according to the GATK best practices for germline WGS data [43]. SVs were analyzed using the FindSV pipeline [44], a pipeline combining CNVnator V0.3.2 [45] and TIDDIT V2.0.0 [46]. The outputs of these callers (~ 27,000 SVs; Additional file 2: Figure S1) are merged using SVDB [47], and the resulting variant calling file (VCF) is annotated using variant effect predictor (VEP) 87 [48]. Finally, the VCF is annotated and sorted based on the allele frequencies in the Swedish structural variant frequency database (SweFreq SVDB) [6, 49], as well as an internal database (internal SVDB) consisting of approximately 400 individuals.

SNVs were called using MIP [34], a pipeline that combines Samtools [50], FreeBayes [51], and the GATK HaplotypeCaller [43] generating an average of 5,500,000 SNVs and INDELS (Additional file 2: Figure S2). Finally, ExpansionHunter v2.5.5 [30] was applied to the Illumina short-read whole-genome alignment files produced by MIP v.6.0.0, assessing STRs in 17 genes (*AR*, *ATN1*, *ATXN1*, *ATXN10*, *ATXN2*, *ATXN3*, *ATXN7*, *C9ORF72*, *CACNA1A*, *CBL*, *CSTB*, *DMPK*, *FMR1*, *FXN*, *HTT*, *JPH3*, and *PPP2R2B*).

### Data analysis of WGS data in the prospective study

The WGS data was analyzed in three steps: (1) large CNVs, (2) small CNVs and genomic rearrangements, and (3) SNVs, INDELS, and repeat expansions. A panel of genes linked to intellectual disability (ID gene panel) that consisted of 887 genes was generated based on the information available at the time through the Genomics England panel app [52] (Additional file 2: Document S1) which was used for filtering small CNVs, SNVs, and INDELS. In addition, for individuals with other clinical symptoms, personalized gene panels were generated based on the patient-specific Human Phenotype Ontology (HPO) [53, 54] terms using the database available through Charité [55] to link the HPO terms and genes.

For SV analysis (aforementioned steps 1 and 2), the output data from FindSV was filtered based on variant size (intergenic variants > 10 kb, and intragenic variants > 2 kb), quality (minimum 6 read pairs (TIDDIT), minimum 5 kb in size (CNVnator)), and allele frequency (SweFreq SVDB < 0.5%, internal SVDB < 1%), and calls located on the decoy contig were also filtered out. Finally, a list of SVs with junctions located within the ID or HPO gene list was generated without a size cutoff. The number of SVs remaining after each filtering step is available in the supplemental data (Additional file 2: Figure S1).

- Step 1: To visualize large CNVs, we used vcf2cytosure [56] that converts a VCF with structural variations to the “.CGH” format used by the CytoSure Interpret Software by OGT. CytoSure is normally used in our laboratory to interpret the clinical significance of CNVs detected by CMA. By displaying the WGS data in a familiar system where we have access to a large internal database of previously assessed cases, variant classification is facilitated (Additional file 2: Figure S3). As described in the CMA section, detected CNVs were classified into five categories according to the ACMG guidelines [57].
- Step 2: Selected SVs were visualized in a list view enabling more detailed assessment of balanced/complex genomic rearrangements. Indications of a genomic rearrangement include (i) several SVs in the same genomic region, (ii) discordant read pairs mapping from a duplication/deletion boundary to another chromosomal region, and (iii) several CNVs clustering on the same chromosome. All such variants of interest were then inspected in IGV, and the derivative chromosomes were reconstructed by visual inspection and breakpoint junction analysis [58].
- Step 3: SNVs and INDELS were annotated using MIP [34] and repeat expansions with ExpansionHunter [30]. Rare variants with a minor allele frequency (MAF) less than 1% in Exome Aggregation Consortium (ExAC) [8, 59] or in the Swedish variant frequency database (SweFreq) [6, 60] and located within the ID or HPO gene list were considered for further analysis. We then used an internal scoring system that also takes into account conservation and predicted severity of the variant [34] that is available on GitHub [61]. The number of SNVs remaining after each filtering step is available in the supplemental data (Additional file 2: Figure S2). Finally, remaining variants were individually assessed and classified according to ACMG guidelines [57] into five classes; benign, likely benign, likely pathogenic, pathogenic, and uncertain significance.

All SNVs and CNVs reported in this study have been submitted to the ClinVar database [62].

Finally, in individual RD_P432, maternal uniparental disomy (UPD) for chromosome 15 was assessed in the WGS data. In brief, this was done in two steps using in-house developed bioinformatic tools. First, we searched for regions of homozygosity on chromosome 15 using rhocall [63] and then we made a run of homozygozity (RoH) plot using the rhoviz tool from the same package. In brief, this assesses zygosity ratios at all SNPs across an entire chromosome (in this case chromosome 15). The zygosity ratio is defined as the ratio of high-quality variants in a local region supporting a heterozygous allele to the total number of high-quality variants in that region.

### Verification of variants identified by WGS

SNVs were verified with PCR and Sanger sequencing. For the two deletions in *MBD5* and *C12orf65* identified by WGS in individuals RD_P416 and RD_P417, respectively, and the reciprocal translocation between chromosomes 4 and 7 (RD_P77) (Table 3), primers flanking the breakpoints were designed approximately 500 base pairs away from the estimated breakpoints. The same primers were subsequently used for sequencing using the Sanger method (primer sequences available upon request). The PCR was performed using standard methods with Platinum Taq DNA Polymerase (Invitrogen, Carlsbad, CA, USA). Sequences were aligned using BLAT (UCSC Genome Browser) [64, 65] and visualized in the CodonCode Aligner software (CodonCode Corp., Dedham, MA, USA).

The *ATXN7* STR identified in individual RD_P431 was verified and the exact number of CAG copies determined using PCR followed by fragment length analysis [66]. The length of the PCR products was determined using capillary electrophoresis on an ABI3500xL Genetic Analyzer and the software Gene Mapper v5 (Applied Biosystems, Thermo Fisher Scientific, Waltham, MA, USA).

Fluorescence *in situ* hybridization (FISH) was performed using standardized protocols from peripheral blood cultures from patient RD_P405, harboring a duplication on chromosome 3 where the WGS data suggested that the duplication had been inserted on chromosome 13. Probes used were RP11-209H21-SG (green, chr3:159,243,721-159,420,409 (Hg19)) and RP11-203L15SO (red, chr3:160,561,956-160,724,921 (Hg19)).

Chromosome analysis was performed on metaphases from peripheral blood cultures from patients RD_P406 and RD_P414 according to standard protocols with subsequent G-banding with an approximate resolution of 550 bands per haploid genome. A total of 12 metaphases were analyzed.

## Results

### WGS reliably identifies deletions, duplications, and aneuploidies and reveals additional clinically relevant genetic information

**Fig. 1:**
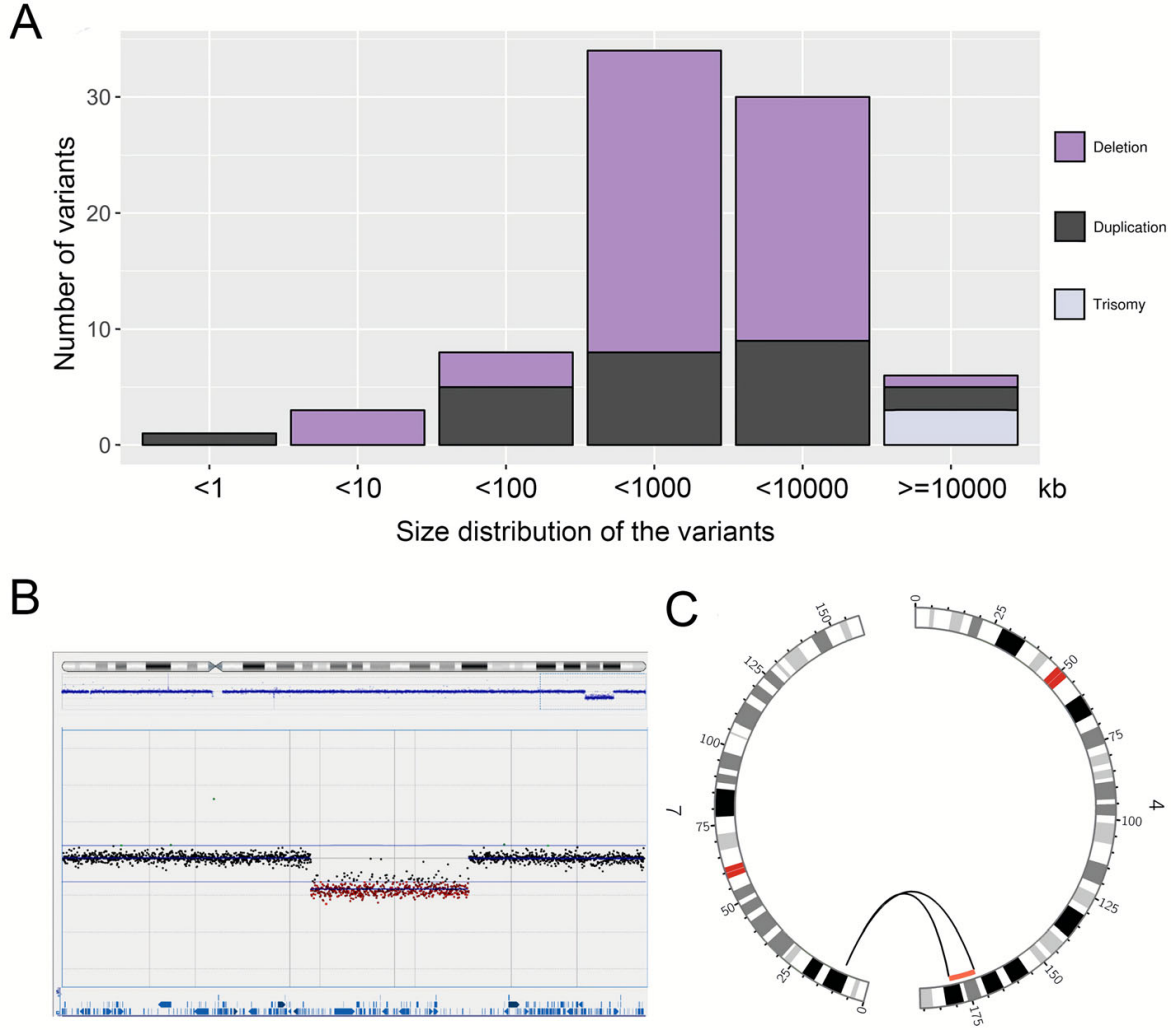
Overview of CNVs and affected individuals included in the validation cohort. **a** Bar graph showing the size distribution of 79 validated CNVs and three trisomies that were detected with WGS. Deletions are shown in purple, duplications in black, and trisomies in lilac. **b** Array comparative genomic hybridization plot indicates a heterozygous deletion of 9.3 Mb in individual RD_P77. **c** Circos plot illustrating the WGS results in the same individual. Discordant read pairs between chromosomes 4 and 7 are shown as gray lines, and the deletion is shown in red

To validate the SV calling pipeline, a total of 68 individuals with three trisomies and 79 CNVs, previously detected by CMA (65 individuals) or MLPA (three individuals), including 54 deletions and 25 duplications, were subjected to short-read WGS sequencing (Fig. 1, Table 2, Additional file 1: Table S1) and analyzed with the FindSV pipeline that includes the two SV callers: CNVnator [45] and TIDDIT [46]. All validation CNVs were detected in the WGS data. The two callers performed slightly differently, mainly depending on whether the CNV breakpoints were located in repetitive regions (Table 2). The size distribution of the variants ranged from 500 bp (single exon CNVs) to 155 Mb (whole chromosome) (Fig. 1, Additional file 1: Table S1).

**Table 2:** Overview of the 80 validated CNVs detected by CMA and WGS in 68 patients

Type	Found by TIDDIT	Found by CNVnator	Found by CMA
Deletions			
Recurrent [7]	0 (0%)	7 (100%)	7 (100%)
Non-recurrent [47]	39 (83%)	44 (94%)	43 (91%)
Total [54]	39 (72%)	51 (94%)	50 (93%)
Duplications			
Recurrent [5]	0 (0%)	5 (100%)	5 (100%)
Non-recurrent [18]	15 (83%)	16 (89%)	15 (83%)
Total [23]	15 (65%)	20 (87%)	21 (91%)
Trisomies			
Total [3]	3 (100%)	3 (100%)	3 (100%)

*CMA* chromosomal microarray, *WGS* whole-genome sequencing

#### Deletions

A total of 54 deletions were identified in cohort 1, including seven recurrent (13%) and 47 non-recurrent (87%) (Table 2). The size ranged between 5 kb and 47 Mb (Fig. 1, Additional file 1: Table S1). TIDDIT identified a total of 39 deletions (83%), all of which were non-recurrent while CNVnator identified a total of 51 deletions (94%), including all recurrent deletions (Table 2). The seven recurrent deletions included four contiguous gene syndromes: DiGeorge syndrome (22q11.21 deletion; MIM 188400), Chromosome 1p36 deletion syndrome (MIM 607872), Chromosome 16p11.2 deletion syndrome (MIM 611913), and Prader-Willi syndrome (MIM 176270) due to a 15q11.2 deletion (Additional file 1: Table S1).

#### Duplications

A total of 23 unique duplications were identified in cohort 1, five of which were recurrent (22%) and 18 were non-recurrent (78%) (Table 2). The size of the duplications ranged between 474 bp and 36.4 Mb (Fig. 1, Additional file 1: Table S1). All five recurrent duplications were detected by CNVnator. The recurrent duplications included two contiguous gene syndromes, the 22q11.21 duplication syndrome (MIM 608363; two individuals) and the 16p11.2 duplication syndrome (MIM 614671) (Additional file 1: Table S1). Two duplications were part of complex rearrangements and are discussed below. Of the 16 unique simple duplications, 13 (81%) were found by TIDDIT, allowing for determination of the positioning of the duplicated segments. It was found that all those duplications were in tandem (Table 2, Additional file 1: Table S1).

#### Trisomies

Three of the individuals in cohort 1 harbored chromosomal aneuploidies, one case with a mosaic trisomy 9 (RD_P167), one with trisomy 21 (RD_P25), and one with trisomy X (RD_P101) (Additional file 1: Table S1). In all cases, the trisomy was detected by CNVnator and verified with the coverage track provided by TIDDIT [46, 67]. In individual RD_P167, the mosaicism level was estimated to be ~ 46% with CMA and karyotyping showed it to be present in ~ 29% (15/51) of analyzed metaphases. The WGS data confirmed the presence of a mosaic trisomy 9 present in ~ 46% of cells.

#### Complex rearrangements

Three rearrangements in the validation cohort were known to be complex from the molecular cytogenetics analysis: RD_P22 (DEL-NML-DEL), RD_P07 (DEL-NML-DEL-NML-DUP), RD_P05 (DEL-DUP-DEL) (DEL; deletion, NML; normal, DUP; duplication) (Additional file 1: Table S1), and in an additional five individuals (5/68, 7%), the WGS analysis identified unexpected complexities compared to the original molecular cytogenetic results. These five unexpectedly complex cases included two DEL-INV-DEL rearrangements (RD_P54, RD_P26) and two DUP-NML-DUP rearrangements (RD_P106, RD_P105). The results from those eight cases have been reported previously in an article focused on the detailed characterization of clustered CNVs [68].

In addition, a 9.3-Mb *de novo* deletion on chromosome 4 turned out to be part of a reciprocal translocation between chromosomes 4 and 7 (RD_P77) (Fig. 1, Additional file 1: Table S1). The reciprocal translocation was confirmed with breakpoint junction PCR that confirmed the presence of both junctions. The breakpoint junction analysis of the rearrangement (t(4;7)(q33;p21.3)) revealed non-templated insertions of 8 and 5 nucleotides in both junctions and no microhomologies, consistent with non-homologous end-joining (NHEJ), the most prevalent formation mechanism underlying reciprocal translocations [27, 69] (Additional file 2: Figure S4).

### Implementation of WGS-SV calling in monogenic rare diseases increases the diagnostic yield

**Fig. 2:**
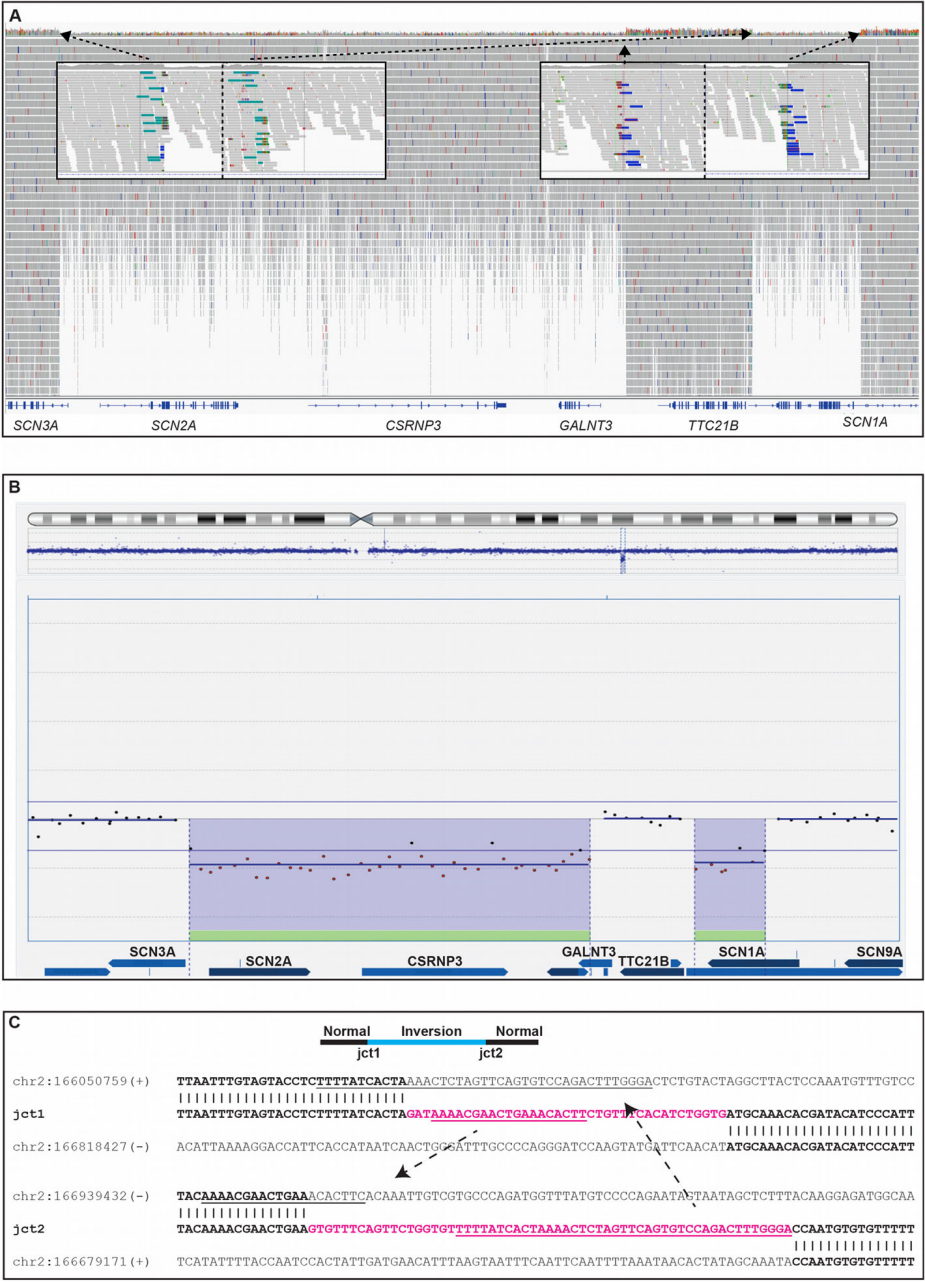
A complex DEL-INV-DEL rearrangement identified by WGS causes severe epilepsy. **a** Screenshot of the deletions and inversion from the Integrative Genomics Viewer (IGV) in individual RD_P393. Short-read whole-genome sequencing (WGS) detected two clustered deletions of 630 kb (*SCN3A*, *SCN2A*, *CSRNP3*, *GALNT3*) and 121 kb (*SCN1A*), respectively. The genomic segment of normal copy number state in-between the deletions (139 kb, *TTC21B*) had been inverted. Both inversion breakpoint junctions are shown with the green and blue bars corresponding to discordant reads with mates located on the other side of the inversion. **b** Screenshot of DEL-INV-DEL rearrangement confirmed by array comparative genomic hybridization (array-CGH). Screenshot from the Cytosure Interpret Software. The deletions in the rearrangement were confirmed using array-CGH. **c** Breakpoint junction sequences. Sequence analysis of the breakpoint junctions revealed insertions in both junctions of 38 bp and 59 bp, respectively (pink). Substantial parts of the insertions had been templated from sequences involved in the rearrangement (underlined), suggestive of a replicative error as the underlying mechanism of formation. L1 repetitive elements were present in two of the breakpoints but did not form any fusion L1 elements. Lowercase letters indicate deleted sequences

Next, we explored the diagnostic yield of gene-focused SV analysis in 156 patients referred for genetic investigation due to a clinical suspicion of monogenic disease. At the Karolinska University Hospital, we have since 2015 used WGS in individuals with a clinical suspicion of monogenic diseases to screen for SNVs and INDELs in multi-gene panels created by *in silico* filtering of the WGS data. Here we sought to implement SV calling focused on variants within the patient-specific *in silico* panel. In this way, we identified 12 clinically relevant SVs (8%): eight deletions, two duplications, one inversion, and one complex rearrangement with two deletions and an inversion (DEL-INV-DEL) (Table 3, Fig. 2) increasing the diagnostic yield.

**Table 3:** Clinically relevant structural variants detected in 156 clinical WGS *in silico* gene panels

Sample ID	Reason for referral	Aberration type	Zygosity	Gene	Coordinates (hg19)	Size (bp)	Classification
RD_P391	SKD	Deletion	Heterozygous*	*DYNC2H1* (exons 19–78)	chr11:103016472-103177263	161,791	Likely pathogenic
RD_P392	Malformations	Deletion	Homozygous	*B9D1* (exon 4)	chr17:19250943-19251153	210	Pathogenic
RD_P393	Epilepsy	Deletion	Heterozygous	*SCN3A* (exon 1*)*, *CSRNP3*, *GALNT3* (whole gene)	chr2:166050817-166679227	628,410	Pathogenic
		Inversion	Heterozygous	*TTC21B* (whole gene)	chr2:166679228-166818452	139,224	
		Deletion	Heterozygous	*SCN1A* (whole gene)	chr2:166818453-166939516	121,063	
RD_P394	NMD	Duplication	Homozygous	*LAMA2* (exon 30)	chr6:129655050-129670080	15,030	Pathogenic
RD_P395	NMD	Duplication	Homozygous	*LAMA2* (exon 30)	chr6:129655050-129670080	15,030	Pathogenic
RD_P396	NMD	Deletion	Heterozygous	*DMD* (exon 45)	chrX:31973924-32017000	43,076	Pathogenic
RD_P397	NMD	Deletion	Hemizygous	*DMD* (exon 3–21)	chrX:32493944-33021034	527,090	Pathogenic
RD_P398	Lissencephaly	Deletion	Heterozygous	*PAFAH1B1* (exon 3–11)	chr17:2555675-2645203	89,528	Pathogenic
RD_P399	NMD	Deletion	Homozygous	*DYSF* (exon 6–11)	chr2:71740967-71749805	8838	Likely pathogenic
RD_P400	SKD	Deletion	Heterozygous	*COPS7B* (whole gene), *NPPC*, *DIS3L2* (exons 1–5)	chr2:232647812-232930068	282,200	Likely pathogenic
RD_P401	Eye disorder	Inversion	Hemizygous	*CHM* (exon 1)	chrX:85296959-85303375	6401	Pathogenic
RD_P402	SKD	Deletion	Heterozygous	*KDM6A* (whole gene)	chrX:44207077-45518941	1.3 Mb	Pathogenic

*SKD* skeletal dysplasia, *NMD* neuromuscular disease. *Heterozygous missense in trans

#### A founder duplication in LAMA2 is the cause of neuromuscular disease in two unrelated individuals

An identical homozygous duplication of exon 30 in *LAMA2* was found in two seemingly unrelated individuals of the same ethnic origin (RD_P394, RD_P395; Table 3) with muscular dystrophy. The duplication was not found in DGV, The Genome Aggregation Database (gnomAD) [70], or the SweFreq SVDB databases. *LAMA2* (located on chromosome 6q22–23) consists of 65 exons and encodes the protein merosin. The WGS data showed signals consistent with a tandem duplication, which conceptually will produce one normal copy of exon 30 followed by a frameshift and a stop codon after 35 aberrant residues. Immunohistochemical antibody staining of merosin in muscle biopsies from both patients showed absence of the protein, consistent with a loss-of-function mutation and conforming to a diagnosis of autosomal recessive muscular dystrophy (MIM 607855) [71].

#### A heterozygous deletion on 2q27 causes dominant skeletal dysplasia

In individual RD_P400 (Table 3), CMA and WGS analysis identified a maternally inherited 282-kb deletion on chromosome 2q27 affecting three genes, the entire *COPS7B*, *NPPC*, and exons 1–5 of *DIS3L2*. The patient had disproportionate short stature, brachydactyly E, and small hands and feet. Her phenotype is summarized in Additional file 2: Document S2. Recently, two unrelated families were reported with heterozygous missense variants in *NPPC* segregating with a short stature and small hand phenotype, very similar to that of our patient and her mother, have been described [72] (Additional file 2: Document S2). Further, *NPPC* plays an important role in endochondral ossification through regulation of chondrocyte proliferation and differentiation in the cartilaginous growth plate [73]*.* It binds and stimulates the cGMP production of the NPR2 receptor and is through that pathway involved in the pathogenesis of relatively common skeletal dysplasias such as achondroplasia (MIM 100800) and acromesomelic dysplasia, Maroteaux type (MIM 602875).

#### A complex intrachromosomal rearrangement disrupting three epilepsy genes

A DEL-INV-DEL rearrangement was identified by WGS in individual RD_P393, a girl with developmental delay and severe epilepsy. A detailed clinical description is available in the supplemental information (Additional file 2: Document S2). Due to a clinical suspicion of monogenic disease, WGS was performed and filtered for genes involved in inborn errors of metabolism as well as epilepsy without identifying a causative genetic variant. The WGS data was then analyzed for structural variants using the FindSV pipeline, and the results showed a *de novo* complex rearrangement on chromosome 2q24.2 including two heterozygous deletions separated by an inverted fragment. The two deletions were visible by CMA and directly affected four genes (*SCN1A*, *SCN2A*, *SCN3A*, and *CSRNP3*) (Fig. 2a, b, Table 4). The proximal deletion was 628 kb, and the proximal breakpoint disrupted intron 1 of *SCN3A* and the distal breakpoint were located 28 kb upstream of *GALNT3*, with a complete heterozygous loss of *GALNT3*, *SCN2A*, and *CSRNP3*. The distal deletion was 121 kb, with the proximal breakpoint 28 kb downstream of *SCN1A* and the distal breakpoint in intron 1 of *SCN1A*. Finally, the 139 kb genomic segment located in-between the deletions had been inverted resulting in a DEL-INV-DEL pattern (Fig. 2). Analysis of the breakpoint junctions revealed partially templated insertions of 38 bp and 59 bp, respectively (Fig. 2c). The insertions did not originate from the sequences in direct proximity of each junction, instead parts of the insertion in junction 1 originated from junction 2 sequences, and parts of the insertion in junction 2 originated from junction 1 sequences (Fig. 2c). One breakpoint disrupted *SCN1A* in intron 1 and another breakpoint disrupted *SCN3A* in intron 1, with no surrounding repetitive sequences. The remaining two breakpoints did not disrupt any protein coding genes but were located in repetitive regions belonging to the L1 family (L1MC4a and L1MA4). No L1 fusion elements were created as a result of the rearrangement.

**Table 4:** Clinically relevant findings in a prospective study of 100 cases referred for CMA

Case	Reason for referral	CNVs (ISCN 2016)	Size	Classification	OMIM diagnosis (inheritance pattern)	Added information by WGS
Fifteen CNVs detected with CMA and WGS						
RD_P403	NDD	arr[GRCh37] 2p21p22.1(39053852_42501893)x3 dn	3.45 Mb	Likely pathogenic	Not in OMIM	Tandem duplication
RD_P404	NDD	arr[GRCh37] 3p25.2p25.3(9453917_12015126)x3	2.56 Mb	Likely Pathogenic	Not in OMIM	Tandem duplication
RD_P405	Growth retardation	arr[GRCh37] 3q25.32q26.1(158567751_160802139)x3 (not paternal)	2.23 Mb	Likely Pathogenic	Not in OMIM	Duplication inserted on chromosome 13
RD_P406	NDD, microcephaly	arr[GRCh37] 4q25q35.2(190816609_191024533)x3 dn	81 Mb	Pathogenic	Not in OMIM	Unbalanced translocation between chr 4 and chr2
RD_P407	NDD, Arthrogryposis	arr[GRCh37] 7q11.23(72699382_74142329)x1 dn	1.44 Mb	Pathogenic	#194050 Williams-Beuren syndrome (AD)	
RD_P408	Short stature, NDD, facial dysmorphism	arr[GRCh37] 7q11.23(72726590_74142329)x1 dn	1.42 Mb	Pathogenic	#194050 Williams-Beuren syndrome (AD)	
RD_P409	Hypogonadotropic hypogonadism	arr[GRCh37] 8p11.22(38320755_38328265)x1 dn (exon 1 *FGFR1*)	7.52 kb	Pathogenic	#147950 Kallmann syndrome (AD)	
RD_P410	Duodenal atresia	arr[GRCh37] 9p24.2p24.3(2074076_2381053)x1 pat	307 kb	VUS	Not in OMIM	
RD_P411	NDD, Arthrogryposis	arr[GRCh37] 12q13.13(53784698_54741363)x1 dn	957 kb	Pathogenic	Not in OMIM	
RD_P412	NDD	arr[GRCh37] 15q13.2q13.3(30405535_32914190)x1	2.5 Mb	Pathogenic	#612001 15q13.3 microdeletion syndrome (AD)	
RD_P413	NDD	arr[GRCh37] 16p11.2(29656717_30158469)x1	502 kb	Pathogenic	#611913 16p11.2 microdeletion syndrome (AD)	
RD_P414	NDD	arr[GRCh37] 18p11.32pter(12774-1652788)x1 dn, 18q22.1qter(62984563_78015117)x1 dn (Ring chromosome)	1.64 Mb 15 Mb	Pathogenic	Not in OMIM	Detailed structure of derivative chromosome
RD_P415	NDD	arr[GRCh37] 22q11.21(18890264_21540347)x1	2.65 Mb	Pathogenic	#188400 DiGeorge syndrome (AD)	
RD_P416	NDD	arr[GRCh37] 2q23.1(148893848_148944832)x1 (exon 3 *MBD5*)	51 kb	VUS	#156200 Intellectual disability 1 (AD)	
One CNV detected with WGS						
RD_P417	NDD	arr[GRCh37] 12q24.31(123736705_123740392)x0 (exon 2 *C12orf65*)	3.7 kb	Pathogenic	#613559 Combined oxidative phosphorylation deficiency 7 (AR)	Deletion found by WGS and not detected by CMA
Case	Reason for referral	SNV	Inheritance	Zygosity	OMIM diagnosis	Classification
Nine SNVs detected with ID *in silico* gene panel						
RD_P418	NDD, macrocephaly	*NSD1*, NM_172349, c.5289G>C, p.(Trp1763Cys)	*De novo*	Heterozygous	#117550 Sotos syndrome (AD)	Likely Pathogenic
RD_P419	NDD (father with similar symptoms)	*NKX2*-1, NM_003317, c.556delC, p.(Leu186CysfsTer12)	Paternal	Heterozygous	#610978 Choreoathetosis, hypothyroidism and neonatal respiratory distress (AD)	Likely Pathogenic
RD_P420	NDD, white matter abnormality	*PNPT1*, NM_033109, c.420delG; p.(Leu141SerfsTer17) *PNPT1*, NM_033109, c.1519G>T, p.(Ala507Ser)	Paternal	Compound heterozygous	#614932 Combined oxidative phosphorylation deficiency 13 (AR)	Likely Pathogenic
			Maternal			
RD_P421	NDD, cleft-lip palate	*SATB2*, NM_015265, c.652A>T, p.(Lys218Ter)	*De novo*	Heterozygous	#612313 Glass syndrome (AD)	Pathogenic
RD_P422	Microcephaly, holoprocencephaly	*ZIC1*, NM_007129, c.1225C>T, p.(Arg409Trp)	*De novo*	Heterozygous	#616602 Craniosynostosis 6 (AD)	Likely Pathogenic
RD_P423	NDD, macrocephaly	*PTEN*; NM_000314, c.264T>G; p.(Tyr88Ter)	N.i.	Heterozygous	#158360 Cowden syndrome (AD)	Likely Pathogenic
RD_P424	Respiratory failure, abnormality of corpus callosum	*POLG*; NM_002693, c.679C>T;679C>T, p.(Arg227Trp)	Paternal/ Maternal	Homozygous	#203700 Mitochondrial DNA depletion syndrome 4A (AR)	Likely Pathogenic
RD_P425	NDD, epilepsy	*CLN5*, NM_006493, c.595C>T;595C>T, p.(Arg199Ter)	Paternal/ Maternal	Homozygous	#256731 Ceroid lipofuscinosis, neuronal 5 (AR)	Pathogenic
RD_P426	NDD, dysmorphic features , macrocephaly	*ANKRD11*; NM_013275, c.3882_3885dupAGAC, p.(Ser1296ArgfsTer5)	N.i.	Heterozygous	#148050 KBG syndrome (AD)	Likely Pathogenic
Four SNVs detected with HPO terms-generated *in silico* gene panel						
RD_P427	NDD, hypotonia, dysmorphic features	*TBCK*, NM_033115, c.469+1G>A;469+1G>A	Paternal/ Maternal	Homozygous	#616900 Infantile hypotonia with psychomotor retardation and characteristic facies 3 (AR)	Pathogenic
RD_P428	Arthrogryposis, dysmorphic features	*ECEL1*, NM_004826, c.494T>C, p.(Leu165Pro) *ECEL1*, NM_004826, c.2228G>T, p.(Arg743Met)	Not maternal	Compound heterozygous	#615065 Distal arthrogryposis type 5D (AR)	VUS
			Maternal			
RD_P429	Hypotonia, hearing loss	*ACOX1*, NM_004035, c.1729-1G>A	Paternal/Maternal	Homozygous	#264470 Peroxisomal acyl-CoA oxidase deficiency (AR)	Likely pathogenic
RD_P430	Hypertrophic cardiomyopathy, severe anemia, respiratory failure	*SPTA1,* NM_003126, c.83G>A, p.(Arg28His) *SPTA1,* NM_003126, c.47854delG, p.(Arg1595Lysfs*38)	Paternal	Compound heterozygous	#270970 Spherocytosis type 3 (AR)	Pathogenic
			Maternal			
Repeat expansions detected by WGS						
RD_P431	Hypotonia, ASD, metabolic acidosis	*ATXN7*, STR	N.i.	Heterozygous	#164500 Spinocerebellar ataxia 7 (AD)	Pathogenic
Uniparental isodisomy validated by WGS						
RD_P432	Hypotonia, dysmorphic features	Maternal UPD 15 (isodisomy)	N.a.	N.a.	#176270 Prader-Willi syndrome (AD)	Pathogenic

*CNV* copy number variant, *CMA* chromosomal microarray, *NDD* neurodevelopmental delay, *WGS* whole-genome sequencing, *VUS* variant of uncertain significance, *SNV* single nucleotide variant, *STR* short tandem repeat, *N.i.* no information, *UPD* uniparental disomy, *N.a.* not applicable, *AD* autosomal dominant, *AR* autosomal recessive

To summarize, this individual is a carrier of a structural variant that leads to loss of function in three distinct Mendelian epilepsy genes. Both deletions and duplications, involving each of *SCN1A*, *SCN2A*, and *SCN3A*, have been presented previously in cases with severe epileptic encephalopathies and developmental delay [74, 75]. In the case presented here, the deletions disrupted *SCN1A* and *SCN3A* and resulted in a complete loss of *SCN2A* (Fig. 2, Table 3, Table 4). The clinical presentation is coherent with Dravet syndrome (MIM 607208), usually caused by mutations involving *SCN1A*; however, the phenotypic variability in *SCN1A* mutation carriers is wide [76]. In addition, mutations in *SCN2A* also cause epileptic encephalopathy (MIM 613721) and missense variants in *SCN3A* have been implicated in focal epilepsy in children [77].

### Prospective study of comprehensive WGS analysis in 100 cases referred for CMA

Finally, we performed a prospective pilot study where the first 100 cases referred to the Department of Clinical Genetics (Karolinska University Hospital, Stockholm, Sweden) for CMA in 2017 were analyzed in parallel with WGS. The obtained WGS data were processed for large SVs (> 10 kb), for genome-wide and small SVs (> 2 kb), and for SNVs and INDELs in 887 genes linked to intellectual disability (Additional file 2: Document S1). For individuals with additional clinical symptoms, custom *in silico* gene panels were created using HPO terms and data were processed for small SVs in the same way as for the ID gene panel. Next, small (> 2 kb) intragenic SVs were assessed in the both gene panels. Finally, we applied the ExpansionHunter analysis pipeline [30] to identify large expansions of STRs in 17 genes.

#### Detection of copy number variants

The CMA analysis identified, in 14 patients, a total of 15 CNVs that were classified as pathogenic (*n* = 10), likely pathogenic (*n* = 3), and variants of uncertain significance (VUS) (*n* = 2) (Table 4). Six rearrangements were recurrent known microdeletion syndromes: 7q11.23 deletions (RD_P407, RD_P408), 8p11.22 deletion (RD_P409), 15q13.2q13.3 deletion (RD_P412), 16p11.2 deletion (RD_P413), and 22q11.21 deletion (RD_P415) while the remainder were non-recurrent/private rearrangements. All of these CNVs were also detected by WGS. In addition, in individual RD_P417, the WGS-SV analysis detected a 3.7-kb homozygous deletion of exon 2 in *C12orf65* confirming a diagnosis of autosomal recessive spastic paraplegia 55 (SPG55) (MIM 615035) (Table 4). This deletion was not called by CMA due to insufficient probe coverage (no probes within the deleted regions).

#### Comparison of WGS-SVs to CNVs detected by CMA

After the array-CGH data was analyzed with standard clinical setting (three consecutive aberrant probes; log2 ratio cutoff − 0.65 for deletions and 0.35 for duplications), a total of 2282 deletions and duplications were called in the 100 patients in cohort 3 (Additional file 3: Table S2). The median number of CNV calls was 22 (quantile (Q)1 18; Q3 26) with a median size of 51 kb (Q1 24 kb; Q3 122 kb). The corresponding numbers from WGS, after filtering for size (> 10 kb intergenic, and > 2 kb intergenic) and frequency (< 0.5% AF in SweFreq SVDB, < 1% in the internal SVDB), were a median number of 28 (Q1 20; Q3 36) and the median size of 17 kb (Q1 6 kb; Q3 70 kb). Comparison of the output data files show that the filtered WGS-SVs only overlap with 9.1% of the CMA calls (208/2282), and if we remove the frequency filter, the overlap increases 34.8% (794/2282) (Table S2). The CMA variants not detected by WGS were smaller, median 38 kb (Q1 19kb, Q3 98kb), compared to those also found with WGS, median 84 kb (Q1 41kb, Q3 154kb). In addition, the percentage of duplications among the CMA variants found by WGS was 52% versus 75% among the variants not found by WGS.

#### WGS reveals the presence of derivative chromosomes and solves their genomic structure

**Fig. 3:**
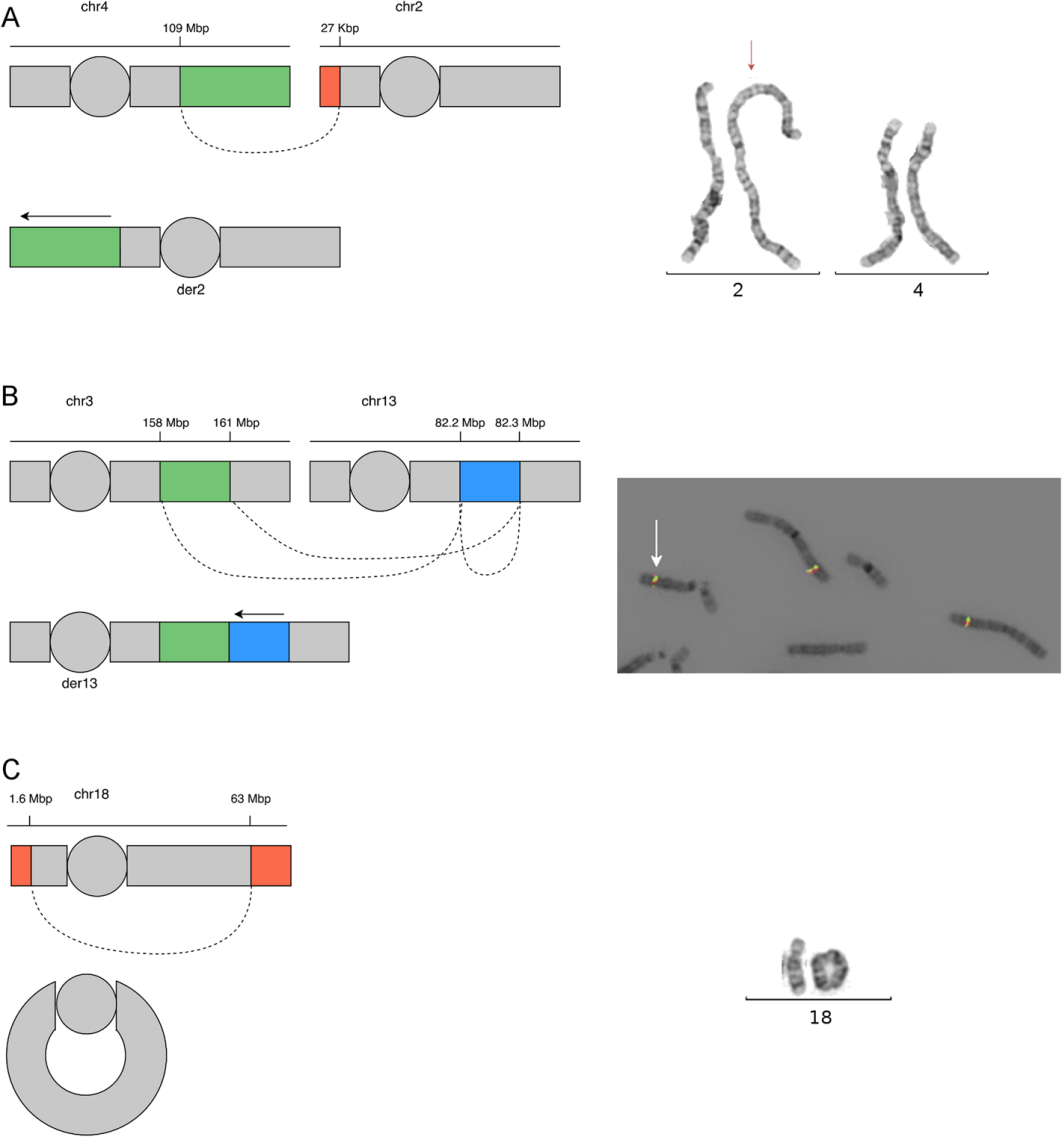
Three cases with complex genomic rearrangements resolved by WGS. **a** A schematic drawing of the 4q25q35.2 unbalanced translocation in individual RD_P406. The duplicated segment of 81 kb (green) is inserted into the p-arm of chromosome 2 directly before the telomeric sequences. A 27-kb deletion on chromosome 2 (orange) is visible in the WGS data. The dashed line represents the links from chromosome 4 to chromosome 2. To the right, the insertional duplication rearrangement is shown through karyotyping with the derivative chromosome 2 indicated by a red arrow. **b** A schematic drawing of the 3q25.32q26.1 insertional duplication in individual RD_P405 as in **a**. The duplicated segment of 2.23 Mb is inserted into chromosome 13, and a genomic segment of 69.6 kb on chromosome 13, adjacent to the insertion, has been inverted. To the right, FISH analysis using probes RP11-209H21SG (green) and RP11-203L15SO (red) located within the rearranged region on chromosome 3. In addition to two signals from chr 3q25.32q26.1, an extra signal is present on chromosome 13 (white arrow) verifying the location of the duplicated segment. **c** A schematic drawing of the r(18) present in individual RD_P414 as in **a**. To the right, the ring chromosome is shown through karyotyping

In case RD_P414, the investigations with CMA identified two deletions on chromosome 18: 1.64 Mb on the terminal p-arm and 15 Mb on the terminal q-arm. Follow-up investigation with chromosome analysis showed that the rearrangement was in fact a ring chromosome present in 100% of the cells. In the same individual, blinded WGS analysis was able to detect the two deletions, characterize the rearrangement breakpoint junctions, and fully resolve the derivative chromosome structure (Fig. 3, Table 4, Additional file 2: Figure S4).

In two cases harboring duplications, the WGS analysis not only detected the presence of a duplication but also revealed that one duplication was part of a cryptic unbalanced translocation and the other was an insertional translocation. In individual RD_P406, an 81-Mb terminal duplication (4q25qter) had been inserted onto the p-arm of chromosome 2 directly before the telomeric sequences. WGS also uncovered a 27-kb deletion on 2p25.3pter not seen by CMA indicating that this was in fact an unbalanced translocation between chromosome 4q and chromosome 2p (Fig. 3, Table 4, Additional file 2: Figure S4). The breakpoint junction had a four-nucleotide non-templated insertion in the junction, and sequence microhomology was low.

In individual RD_P405, a 2.23-Mb duplication of 3q25.32q26.1 was inserted into chromosome 13 (13q31.1). In addition, it was found that there were two breakpoints on chromosome 13, the genomic segment of 69.6 kb between them had been inverted, and the duplication originating from 3q25.32q26.1 was inserted into the proximal breakpoint junction. This was only detected through WGS (Fig. 3). A total of three breakpoint junctions were identified in this patient, and the junctions showed little to no microhomology, no insertions, and no deletions (Table 4, Additional file 2: Figure S4).

Taken together, the breakpoint junctions from both patients harboring insertional duplications revealed no evidence for DNA replication errors, which has been the proposed mechanism underlying the formation of duplications in several cases [68, 78, 79].

In summary, three out of 100 individuals (3%) with intellectual disability referred for CMA carried derivative chromosomes with additional complexities detected and resolved by WGS.

#### Single nucleotide variation

The WGS data was next processed for SNVs and INDELs in a panel of 887 genes (Additional file 2: Document S1) as well as in custom panels created from HPO terms matching the individual patients’ phenotypes. This way, heterozygous SNVs implicated in autosomal dominant disease were identified in six patients (6%), of which five were classified as likely pathogenic and one as pathogenic. Compound heterozygous or homozygous SNVs implicated in autosomal recessive disease were identified in seven patients (7%), of which one was classified as VUS, three as likely pathogenic and three as pathogenic. No X-linked variants were identified (Table 4).

#### WGS diagnoses a lethal form of ataxia type 7 through a repeat expansion in ATXN7

**Fig. 4:**
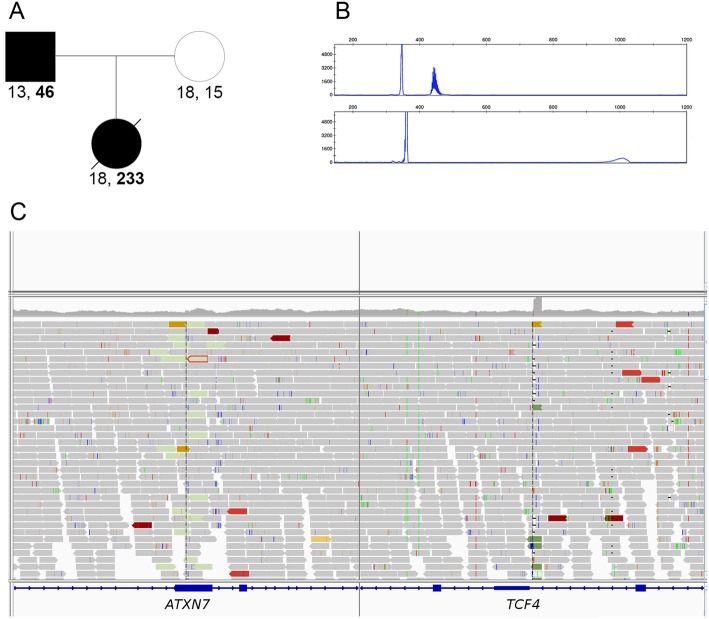
A short tandem repeat expansion in *ATXN7* is identified by WGS. **a** The pedigree and number of *ATXN7* CAG repeats are illustrated under each individual. **b** The PCR-amplified CAG-repeat data from the father shows one normal sized allele and one expanded allele (top chromatogram). In the bottom chromatogram, the results from the affected child are shown. **c** Integrative Genomics Viewer (IGV) screenshot of the data obtained from FindSV shows the first indication of an *ATXN7* abnormality. The aberrant signal was initially interpreted by the program as an insertion of sequence from chromosome 18 (right) into *ATXN7* (left)

Next, we assessed the presence of STRs in 17 genes. A likely pathogenic repeat expansion in *ATXN7* was identified in individual RD_P431 (Table 4). In brief, this was the second child born to unrelated parents. She was treated in the intensive care unit due to hypotonia, cardiac failure, and metabolic acidosis and died at the age of 10 months. The father had poor vision and balance problems but no molecular diagnosis. The detailed clinical description is available in the supplemental information (Additional file 2: Document S2). WGS was performed and filtered for genes involved in inborn errors of metabolism without identifying a causative genetic variant. Upon reanalysis of this case, no suspected pathogenic SNV, INDEL, or SV was identified. However, both FindSV (TIDDIT) and ExpansionHunter indicated an aberrant signal from the *ATXN7* locus. The presence of a CAG STR was confirmed using PCR with fragment length analysis including triplet primed PCR. The detected number of repeated CAG units was 233 in individual RD_P431 and 46 in the father confirming the diagnosis of Spinocerebellar ataxia 7 (SCA7) in both (MIM 164500) (Fig. 4).

#### Maternal isodisomy is visible in the WGS data

**Fig. 5:**
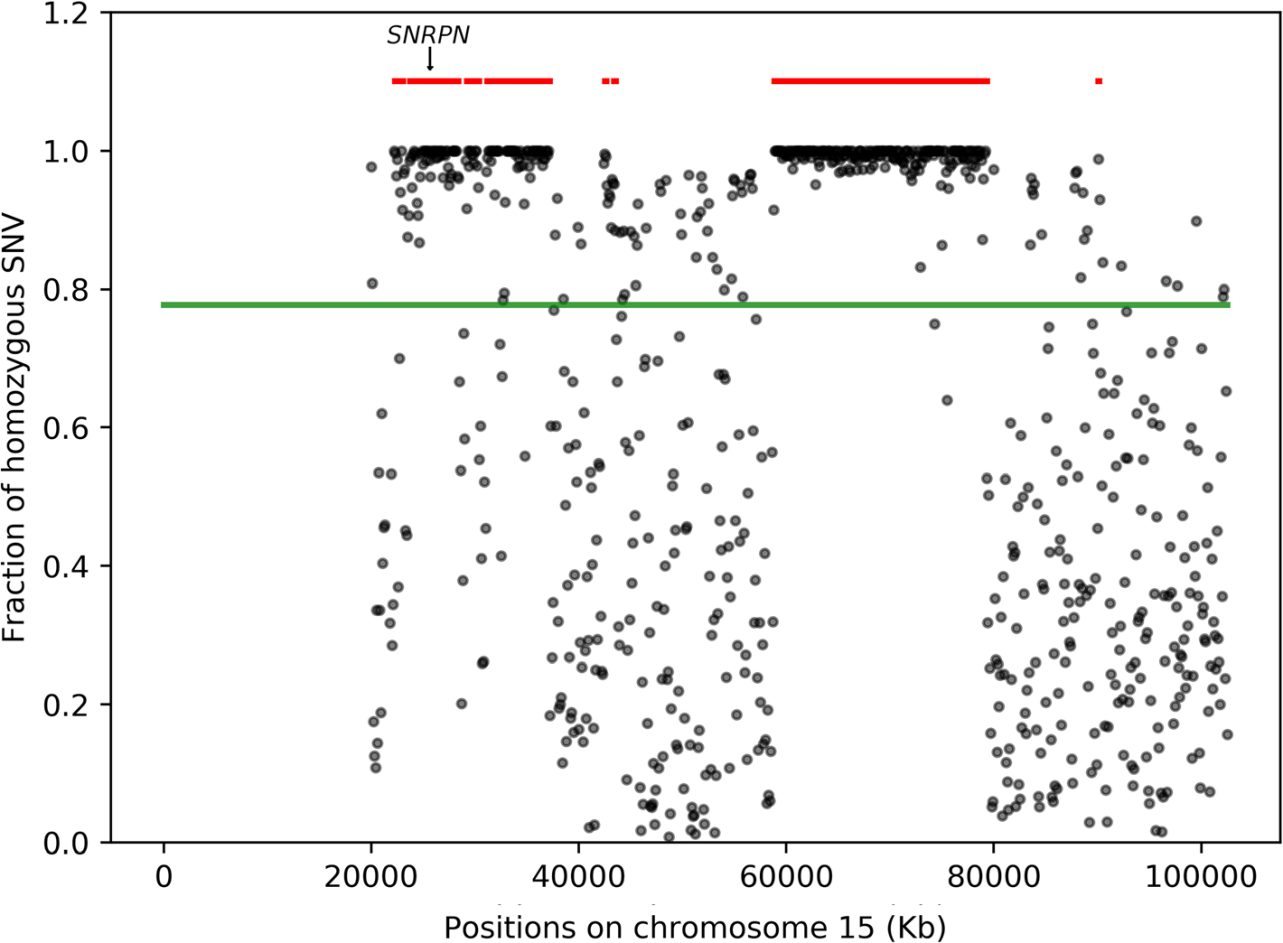
Prader-Willi syndrome caused by maternal isodisomy. Homozygosity for SNPs on chromosome 15 from WGS data in individual RD_P432. The fraction of homozygous SNPs is shown on the Y axis and the position on chromosome 15 on the X axis. The position of *SNRPN* is indicated with an arrow. Each gray dot represents the fraction of homozygous SNVs in 10 kb regions. The green line indicates the fraction of homozygous SNV across the entire chromosome, and red lines indicate autozygous regions predicted by rhocall

Genomic DNA from individual RD_P432 (from cohort 3) was analyzed with methylation-specific MLPA that showed a methylation pattern in the 15q11.2q13 region consistent with a diagnosis of Prader-Willi syndrome (MIM 176270). Genotyping of 10 polymorphic markers located across chromosome 15 (from 22.9 to 49.3 Mb) in the patient and her mother suggested the presence of chromosome 15 maternal uniparental disomy (UPD) for the region 22.9–33.7 Mb. We were able to confirm this finding by assessing the B-allele frequency of SNPs on chromosome 15 in the WGS data. This analysis also confirmed that this was a case of segmental isodisomy (Fig. 5, Table 4).

**Fig. 6:**
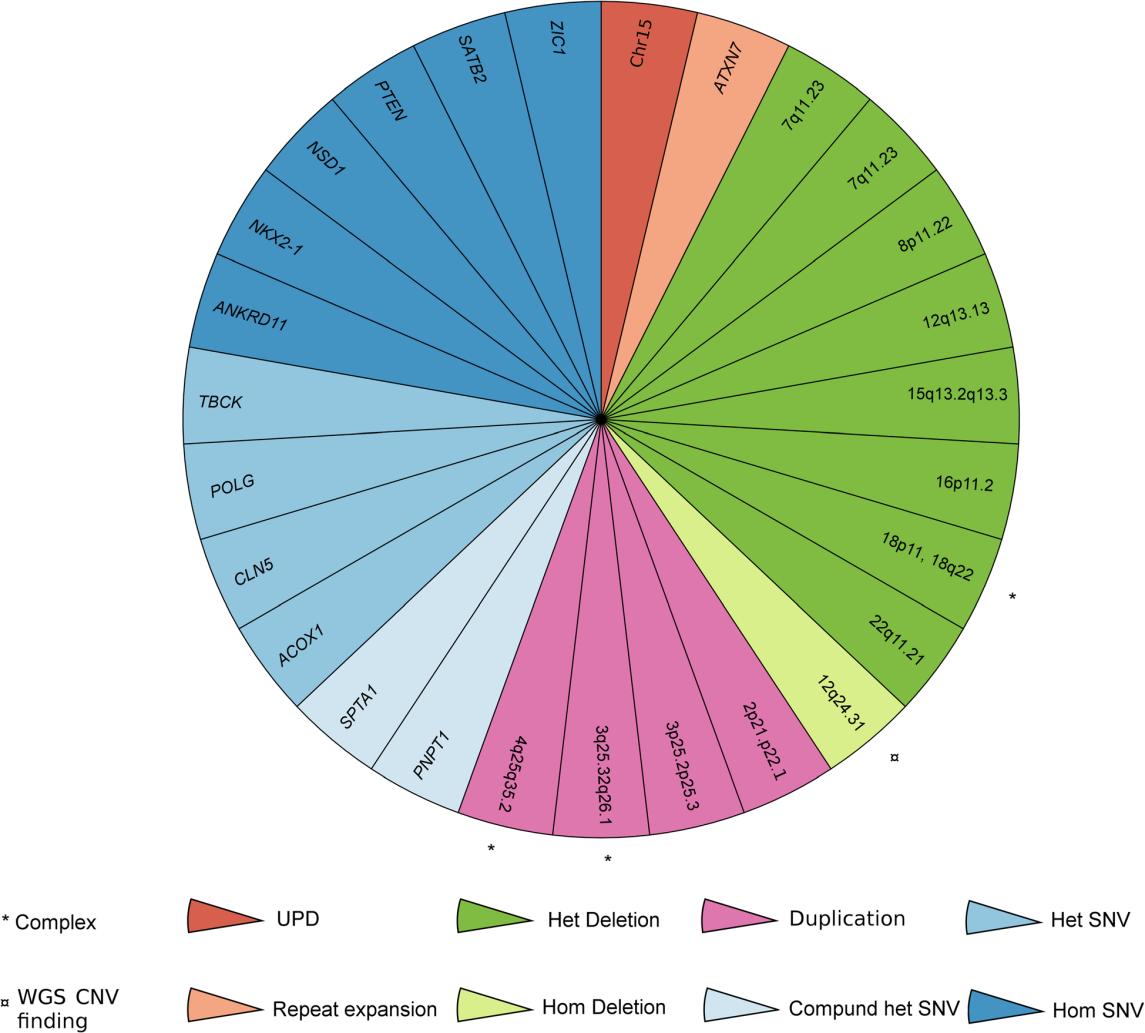
Genetic architecture of a mixed cohort referred for diagnostic analysis. Each slice of the pie chart represents one individual in the 100 prospective cases analyzed by both chromosomal microarray (CMA) and whole-genome sequencing (WGS) where a causal genetic variant was identified. Type of variants is indicated by colors (UPD, red; repeat expansion, orange; homozygous deletion, light green; heterozygous deletion, dark green; duplication, purple; compound heterozygous SNV, light blue; homozygous SNV, blue; heterozygous SNV, dark blue). Additional complexity is indicated by a * and CNVs detected by WGS first with a ¤

Excluding the variants classified as VUS, the diagnostic yield in 100 unselected cases referred for CMA was 27% compared to 12% with array only (Fig. 6).

## Discussion

Previous studies have suggested that short-read WGS could be a first-level test in individuals with neurodevelopmental delay and intellectual disability [80]. Here we show that WGS with regular coverage (30×) indeed captures the complex genetic variation underlying rare diseases in an unselected mixed cohort referred for genetic testing. Altogether, we found that 27% of these individuals harbored clinically relevant genetic variants that could be detected by WGS. In addition, the WGS analysis provided clinically important information by resolving the structure of the derivative chromosomes and revealing additional complexities.

Our data demonstrates that short-read WGS performs well for CNV detection from small intragenic variants to large chromosomal rearrangements. By applying multiple callers with complementary characteristics [45, 46], we successfully detected all 92 known SVs in our three cohorts as well as 11 previously undetected events. We previously compared the performance of the SV callers TIDDIT [46], Manta [81], CNVnator [45], DELLY [82], Fermikit [83], and Lumpy [84] and found that TIDDIT and CNVnator are complementary and offer high precision and sensitivity on large SV [46]. This was confirmed by a recent evaluation of the performance of 69 SV detection algorithms including TIDDIT and CMVnator [85]. Therefore, we chose to combine the TIDDIT and CNVnator callers, enabling the detection of a wide range of variants while using a limited amount of computational resources. Overall, CNVnator [45] failed to detect five aberrations: three deletions (RD_P62, RD_P63, RD_P64) and two duplications (RD_P123, RD_P139) all below 8 kb in size (Additional file 1: Table S1). This highlights that the coverage analysis has limitations in detecting such small CNVs. In contrast, TIDDIT [46] successfully identified all those small CNVs but were unable to call the recurrent events (*n* = 16). This is explained by the mechanism underlying the formation of those rearrangements, non-allelic homologous recombination between repeat structures flanking the CNV. Hence, the breakpoint junctions cluster in those repeats, and since TIDDIT uses split reads and discordant pairs to call an event, the read length (151 bp) and insert size (~ 350 bp) of short-read WGS are not enough to bridge the repetitive regions.

Of note, effective SV screening from WGS is highly dependent on the availability of high-quality frequency databases representing population background variation (and sequencing artifacts) which are essential for the selection of rare potentially disease-causing variants. We filtered the data against our internal database of ~ 400 individuals as well as the SV database from the 1000 Swedish genomes [6] that were analyzed with the same bioinformatic tools used here. After filtering for size (> 10 kb genome wide and > 2 kb in target genes) and frequency (SweFreq SVDB < 0.5%, internal SVDB < 1%), we ended up with a manageable number of SV calls (median 38, average 54; standard deviation 48) that were moved forward to clinical interpretation. The standard deviation could be explained by some individuals of non-Swedish origin and sequencing quality.

The other main obstacle hampering the utility of WGS for SV screening in routine clinical diagnostics is visualization of the structural variants called by WGS. To overcome this, we developed vcf2cytosure [56], a program that converts VCF files with structural variations to the “.CGH” format. The output files are subsequently uploaded into the CytoSure Interpret Software, generally used for visualization of array-CGH data at our clinic (examples of output data visualized in this way are shown in the supplemental data; Additional file 2: Fig. S3). Through this approach, we facilitated the clinical interpretation of the WGS-SV data by non-bioinformaticians.

The 15 additional diagnoses provided by WGS compared to CMA included one homozygous exonic deletion, one STR, one UPD, and 12 SNVs (six heterozygous, four homozygous, and two compound heterozygous) (Table 4). In all cases, obtaining a diagnosis will have an immediate impact by providing more accurate information to the 15 families, enabling both carrier detection and prenatal testing. Embryo diagnostics will be possible for the nine couples with a high recurrence risk; seven with autosomal recessive cause of disease and two with autosomal dominant. In several cases, the molecular diagnosis leads to improved patient care and management, such as the father of RD_P431 with SCA7, and for others, the number of necessary hospital visits was decreased.

In aggregate, our data show that WGS has the potential to be a single test for the detection of the many different genetic variation types underlying rare diseases. However, for each variant class (SVs, SNVs, STRs, ROH, and mosaic events), it is important to understand the limitations of the test. For the calling of SNVs and INDELs, WGS has already shown high sensitivity and specificity [86], but both SVs, STR, and ROH analysis need to be further studied in larger cohorts. Hence, due to a lack of data on sensitivity and specificity of WGS for calling SVs, STRs, and ROH, it is important to remember that a normal result is still hard to interpret and we suggest that all such cases are clinically assessed and if necessary, offered additional testing.

Our evaluation here of WGS-SV analysis in a clinical setting has provided several important insights. First, the detection of 107 disease-causing CNVs (Table 2, Table 3, Table 4, Additional file 1: Table S1) illustrates that WGS can be used to detect disease-causing CNVs of different sizes and types. Second, comparing the WGS-SVs in cohort 3 to all the CNVs called by CMA in the same individuals, we find that the two methods not only produce different amounts of variants but also that only one third of the polymorphic CNVs called by CMA are present in the WGS files (Additional file 3: Table S2). This is problematic, however, we cannot be entirely sure as to which calls are true, since the WGS data is compared to array-CGH data which is derived from relative quantification. Furthermore, the CMA calls not detected by WGS are smaller (median 38 kb vs 84 kb for CNVs detected by WGS) and prone towards duplications (75% vs 52% in CNVs detected by WGS) supporting the notion that these are indeed technical artifacts in the CMA data.

The data presented here also illustrate that screening for STRs and ROH using WGS is a feasible and fruitful approach increasing the number of patients with a definite molecular diagnosis. We consider it clinically relevant to perform these analyses in patients with unexplained intellectual disability. Clinical labs already perform targeted testing for the *FMR1* repeat expansion (FRAX, MIM 309548) and UPD 15 (Angelman syndrome, MIM 105830; Prader-Willi syndrome, MIM 176270) in such cases. If this information is obtainable in the WGS data, it should also be analyzed.

The mosaic trisomy 9 in individual RD_P167 show similar levels of mosaicism by CMA and WGS, and the discrepancy with FISH is likely a culture artifact. The sensitivity of WGS to detect mosaicism still needs to be further evaluated and will most likely depend on the coverage, the caller(s) used, and the specific chromosome affected. Both size and quality of the reference genome as well as GC content will most likely influence the performance. In a previous publication, we assessed the detection rate of a simulated trisomy X and show that it can be detected confidently down to 10% with 30× WGS [67].

The diagnostic yield obtained here (27%) using WGS as a first-tier diagnostic test should be compared to previous studies obtaining over 30% diagnostic yield after SNV analysis of WES data [17]. Since WGS is free from the capture biases that may affect WES and offers the possibility to detect more types of variants (i.e., balanced chromosomal rearrangements, small CNVs affecting single exons, repeat expansions, and deep splice variants), one might expect a higher diagnostic yield. However, the diagnostic yield of WES may range from 8 [87] to 60% [88], depending on the selection criteria and whether proband-only or familial WES was performed [15]. Here we studied a randomly selected cohort of 100 individuals referred for CMA to our center and we only sequenced the probands. After this first screening analysis, the negative cases with a high probability of a genetic cause may be offered trio WGS analysis. In this way, we expect the diagnostic yield to rise, mainly due to an increased detection rate of *de novo* missense mutations in known disease-causing genes but also due to a higher power to detect variation in new undescribed disease-causing genes as well as in non-exonic regions of established genes.

As has been shown previously [29, 78, 89, 90], our data confirms the notion that structural variants are important contributors also to Mendelian diseases (12/156, 7.7%). The *LAMA2* duplication identified in RD_P394 and RD_P395 may represent a founder mutation. As we assess structural variation in more individuals, both healthy and clinically affected, the true frequency of rare founder SVs will also be revealed. This is important and needs to be taken into consideration as we transition to whole-genome diagnostic sequencing.

Several cases in the three studied cohorts harbored known or unexpected complex structural variants. The high accuracy of WGS allowed us to map the breakpoint junctions with nucleotide resolution and study mutational signatures. In the validation cohort, in addition to eight cases of complex intrachromosomal rearrangements that were reported previously [68], a 9.3-Mb de novo deletion on chromosome 4 detected in individual RD_P77 was in fact part of a reciprocal translocation between chromosomes 4 and 7 (Fig. 1; Additional file 1: Table S1, Additional file 2: Document S2). The breakpoint junction analysis revealed no microhomology and non-templated insertions of random nucleotides, indicative of NHEJ repair of double-strand breaks. The complex 2q24.3 rearrangement involving three Mendelian epilepsy genes identified in individual RD_P393 (Fig. 2, Table 3) showed templated insertions in both breakpoint junctions, causing two small segments of 11 bp and 13 bp each to be duplicated. The mutational signatures suggest a replicative error as the mechanism of formation, such as fork stalling and template switching (FoSTeS) [91]. Finally, in the 100 individuals included in the prospective cohort, one ring chromosome (RD_P414) one unbalanced translocation (RD_P406), and one insertional translocation (RD_P405) were resolved (Fig. 3). Each one of the three cases is of a distinct type, and no conclusive underlying mechanism was highlighted from the breakpoint junction analysis.

The ability to outline structural rearrangement connectivity pictures also adds support for the use of WGS as a first-line test in intellectual disability. We have shown previously that this information is important for a proper interpretation of intragenic duplications [78] as well as complex genomic rearrangements (CGRs) [68]. CGRs were a common finding in all three cohorts (8/68; 1/156; 3/100) highlighting that in cases with a genetic disease caused by an SV, the probability of detecting a complex rearrangement is substantial; 12/96 SVs were detected in all three cohorts (12.5%) (Table 2, Table 3, Table 4, Table 5). In a cohort of 100 patients with intellectual disabilities not previously studied by any technique, WGS detected derivative chromosomes accompanied by additional complexities in 3%. Even in individuals with clinical symptoms not expected to be caused by CNVs (such as the cases in cohort 2), the probability is not negligible (0.6%). This type of information would not be provided by most other technologies including CMA.

**Table 5:** Complex rearrangements detected in the current study

Case	Cohort	Type	Chromosome(s)	Phenotype
RD_P22	Cohort 1	DEL-NML-DEL	5	NDD
RD_P54	Cohort 1	DEL-INV-DEL	17	NDD
RD_P07	Cohort 1	DEL-NML-DEL-NML-DUP	1	NDD+
RD_P05	Cohort 1	DEL-DUP-DEL	2	Internal malformations
RD_P26	Cohort 1	DEL-INV-DEL	21	NDD+
RD_P105	Cohort 1	DUP-NML-DUP	7	NDD
RD_P106	Cohort 1	DUP-NML-DUP	14	NDD
RD_P77	Cohort 1	DEL-T	4, 7	NDD+
RD_P393	Cohort 2	DEL-INV-DEL	2	Epilepsy
RD_P405	Cohort 3	DUP-INS	3, 13	Growth retardation
RD_P406	Cohort 3	DUP-INS	4, 2	NDD, microcephaly
RD_P414	Cohort 3	Ring chromosome	18	NDD

*DEL* deletion, *NML* normal, *INV* inversion, *T* translocation, *INS* insertion, *NDD* neurodevelopemental disorder, *NDD+* syndromic NDD

*De novo* CGRs, which may be seen as two or more *de novo* CNVs in the genome, can be observed in ~ 2% of patients with clinical indication to undergo array studies [92] and are more common in some loci associated with genomic disorders. In *MECP2* duplication syndrome (MIM 300260) at Xq28 [93, 94] and Pelizaeus-Merzbacher disease (MIM 312080) due to increased *PLP1* copy number at Xq22 [94–96], specific CGRs account for up to 30% of the pathological SVs. In autosomal *loci*, those CGRs represent a lower number of pathological SVs (< 20%) [97, 98], although technical ascertainment may explain lower detection. Even apparently “simple” non-recurrent rearrangements may actually consist of complex breakpoint junctions formed by multiple insertions of short templated segments (< 100 nucleotides), which was shown in 27 to 35% of simple CNV junctions in disease-associated loci as well as polymorphic CNVs [99]. Moreover, inversions, which cannot be detected by arrays, are associated with CGRs in 84 [100] to 100% of the cases [94, 95, 99].

## Conclusions

In conclusion, our data show that WGS robustly not only captures SNVs but also performs well for the detection of disease-causing CNVs and has the potential to detect STRs, ROH, and chromosomal rearrangements. These findings demonstrate that WGS may be used as a single test instead of performing two separate analyses to detect SVs and SNVs, such as CMA followed by WES, in addition to targeted analyses for specific repeat expansions and UPDs. Even though further studies are necessary to fully understand the limitations of WGS and how to interpret a normal result, for clinics already using clinical WGS for SNV analysis, the added value of mining the data for additional types of disease-causing mutations is high.

## Supplementary information

**Additional file 1:** **Table S1.** Detailed information of individuals in Cohort 1.

**Additional file 2:** **Figure S1.** Flowchart showing different filtering steps of WGS SV analysis, **Figure S2.** Flowchart showing different filtering steps of WGS SNV analysis. **Document S1.** List of genes in the intellectual disability panel. **Figure S3.** Vcf2cytosure and array plots of illustrative cases. **Figure S4.** Breakpoint junction analysis of individuals RD_P77, RD_P393, RD_P400 and RD_P431. **Document S2.** Detailed clinical descriptions of individuals RD_P77, RD_P393, RD_P400 and RD_P431.

**Additional file 3:** **Table S2.** Number of detected CNVs using array and WGS.

## Data Availability

The datasets supporting the conclusions of this article are included within the article and its additional files. All variants reported have been submitted to ClinVar [62], accession number SCV000897707 (*ATXN7* expansion) and submission number SUB5433665. The consent provided by the research subjects did not permit sharing of the entire genome-wide data set. The in-house databases used in this article also contain information from clinical samples and are not publicly available due to compromise of patient confidentiality. The following public databases and open source software were used: Genome Reference Consortium Human Build 37 (https://www.ncbi.nlm.nih.gov/assembly/GCF_000001405.13/) [36]. The Swedish variant frequency database (SweFreq) [60] and the Swedish structural variant frequency database (SweFreq SVDB) [49], both available from https://swefreq.nbis.se/ [6]. The Human Phenotype Ontology (HPO) term database (http://compbio.charite.de/hpoweb/) [55]. The Genomics England panel app (https://panelapp.genomicsengland.co.uk/) [52]. The ClinVar database (https://www.ncbi.nlm.nih.gov/clinvar/) [62]. The Online Mendelian Inheritance in Man (OMIM; https://www.omim.org) [40]. The University of California Santa Cruz (UCSC) Genome Browser (www.genome.ucsc.edu) [65]. The Database of Genomic Variants (DGV; http://dgv.tcag.ca) [38]. The Database of Chromosomal Imbalance and Phenotype in Humans using Ensembl Resources (DECIPHER; http://decipher.sanger.ac.uk) [39]. Exome Aggregation Consortium (ExAC v0.2; http://exac.broadinstitute.org/) [59]. The Genome Aggregation Database (gnomAD; https://gnomad.broadinstitute.org/) [70]. FindSV pipeline (https://github.com/J35P312/FindSV) [44]. SVDB (https://github.com/J35P312/SVDB) [47]. FreeBayes (https://arxiv.org/abs/1207.3907) [51]. vcf2cytosure (https://github.com/NBISweden/vcf2cytosure) [56]. rhocall (https://github.com/dnil/rhocall) [63].

## Abbreviations

WGS: Whole-genome sequencing
SNV: Single nucleotide variant
CNV: Copy number variant
SV: Structural variant
HPO: Human Phenotype Ontology
STR: Short tandem repeat
INDEL: Small insertions and deletions
MPS: Massively parallel sequencing
CMA: Chromosomal microarray analysis
FISH: Fluorescent in situ hybridization
WES: Whole-exome sequencing
UPD: Uniparental disomy
array-CGH: Array comparative genomic hybridization
MLPA: Multiplex ligation-dependent probe amplification
ACMG: American College of Medical Genetics and Genomics
MAF: Minor allele frequency
ExAC: Exome Aggregation Consortium
RoH: Run of homozygozity
NHEJ: Non-homologous end-joining
VUS: Variants of uncertain significance
CGR: Complex genomic rearrangement
F: Female
M: Male
NDD: Neurodevelopmental disorder
CTD: Connective tissue disorder
SKD: Skeletal dysplasia
NMD: Neuromuscular disease
