# Supplementary material for: From cytogenetics to cytogenomics: whole-genome sequencing as a first-line test comprehensively captures the diverse spectrum of disease-causing genetic variation underlying intellectual disability
Source: Genome Med. 2019 Nov 7;11:68. doi: 10.1186/s13073-019-0675-1 (PMC6836550; doi:10.1186/s13073-019-0675-1)
Supplement: Supplementary file 2 — Additional file 2: Figure S1. Flowchart showing different filtering steps of WGS SV analysis, Figure S2. Flowchart showing different filtering steps of WGS SNV analysis. Document S1. List of genes in the intellectual disability panel. Figure S3. Vcf2cytosure and array plots of illustrative cases. Figure S4. Breakpoint junction analysis of individuals RD_P77, RD_P393, RD_P400 and RD_P431. Document S2. Detailed clinical descriptions of individuals RD_P77, RD_P393, RD_P400 and RD_P431. [file 13073_2019_675_MOESM2_ESM.pdf]

Figure S1: Flowchart showing the different filtering steps of WGS SV analysis

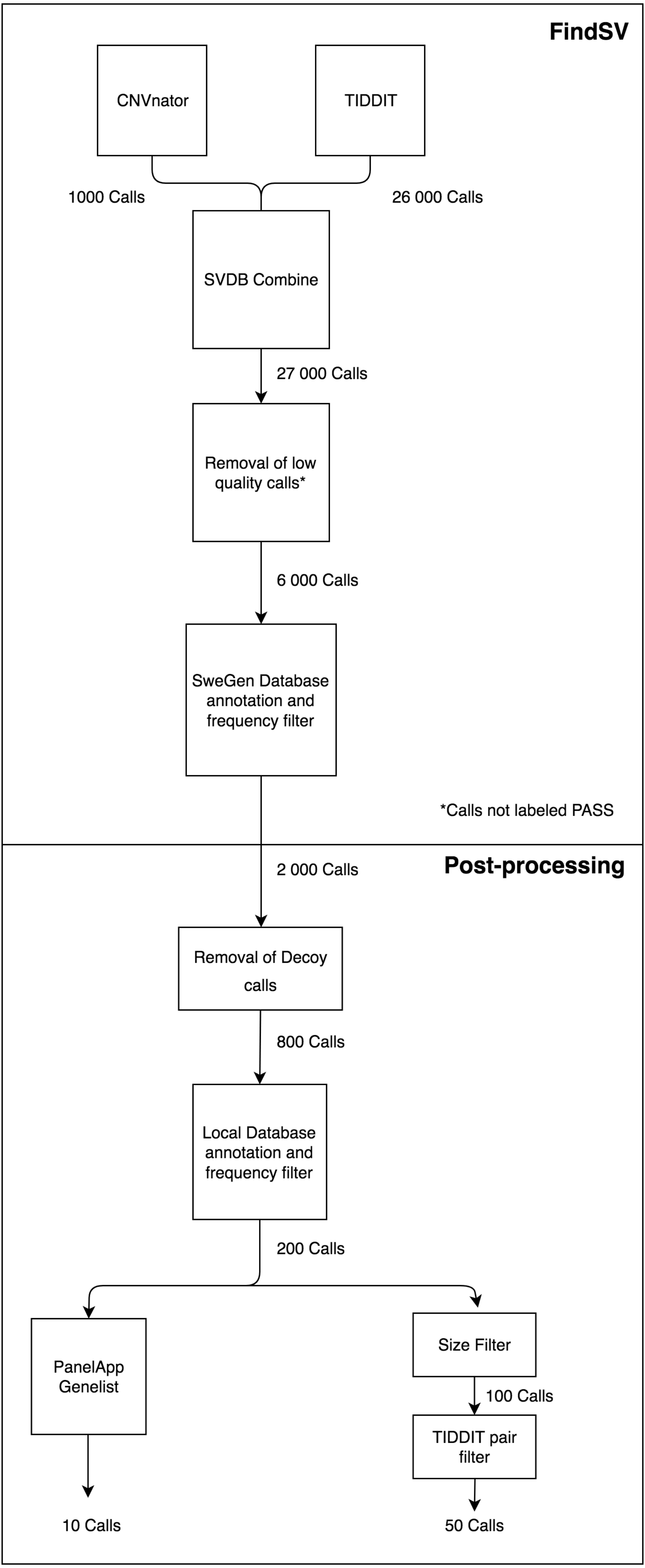

Figure S2: Flowchart showing the different filtering steps of WGS SNV analysis

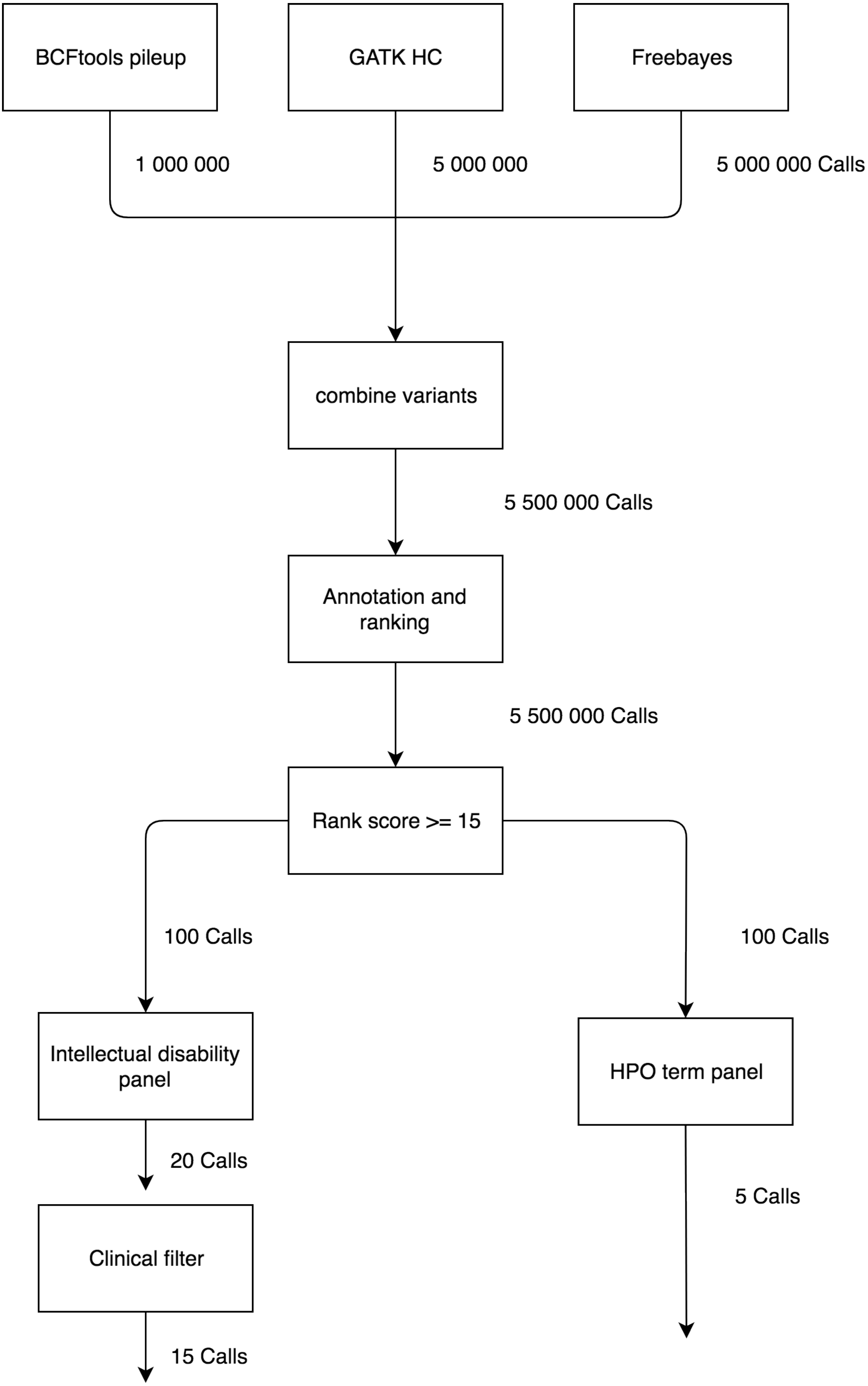

Document S1: List of genes in the intellectual disability panel

PANEL OF 887 GENES LINKED TO INTELLECTUAL DISABILITY

AAAS, AARS, AASS, ABCC9, ABCD1, ABCD4, ABHD5, ACAD9, ACADM, ACADS, ACO2, ACOX1, ACSL4, ACTB, ACTG1, ACTL6A, ACY1, ADAR, ADGRG1, ADK, ADNP, ADSL, AFF2, AFF4, AFG3L2, AGA, AGPS, AHDC1, AHI1, AIFM1, AIMP1, AKT3, ALDH18A1, ALDH3A2, ALDH4A1, ALDH5A1, ALDH7A1, ALG1, ALG11, ALG12, ALG13, ALG3, ALG6, ALG8, ALMS1, ALS2, AMER1, AMPD2, AMT, ANKH, ANKRD11, AP1S2, AP3B1, AP3B2, AP4B1, AP4E1, AP4M1, AP4S1, APOPT1, APTX, ARFGEF2, ARG1, ARHGEF6, ARHGEF9, ARID1A, ARID1B, ARID2, ARL13B, ARL6, ARMC9, ARSA, ARSB, ARSE, ARX, ASAH1, ASL, ASPA, ASPM, ASXL1, ASXL2, ASXL3, ATAD3A, ATIC, ATM, ATP13A2, ATP1A3, ATP6V0A2, ATP6V1B2, ATP7A, ATR, ATRX, AUH, AUTS2, B3GALNT2, B3GLCT, B4GALNT1, B4GALT7, BBS1, BBS10, BBS12, BBS2, BBS4, BBS5, BBS7, BBS9, BCAP31, BCKDHA, BCKDHB, BCL11A, BCOR, BCS1L, BLM, BMP4, BOLA3, BRAF, BRAT1, BRPF1, BRWD3, BSCL2, BTD, BUB1B, C12orf57, C12orf65, C5orf42, CA2, CA8, CACNA1A, CACNA1C, CACNA1D, CAMK2A, CAMK2B, CAMTA1, CASK, CBL, CBS, CC2D1A, CC2D2A, CCBE1, CCDC22, CCDC8, CCDC88C, CCND2, CDC6, CDH11, CDH15, CDK13, CDK5RAP2, CDKL5, CDON, CDT1, CENPF, CENPJ, CEP135, CEP152, CEP290, CEP41, CEP57, CEP83, CHAMP1, CHD2, CHD4, CHD7, CHD8, CHMP1A, CIC, CISD2, CIT, CKAP2L, CLCN4, CLN3, CLN5, CLN6, CLN8, CLP1, CLPB, CLTC, CNKSR2, CNNM2, CNTNAP2, COASY, COG1, COG4, COG5, COG7, COG8, COL4A1, COL4A2, COL4A3BP, COLEC11, COQ4, COQ8A, COX10, COX15, COX6B1, CPS1, CRADD, CRB2, CREBBP, CSNK2A1, CSPP1, CSTB, CTC1, CTCF, CTDP1, CTNNB1, CTSA, CTSD, CUL4B, CYB5R3, CYC1, CYP2U1, D2HGDH, DAG1, DARS, DARS2, DBT, DCAF17, DCHS1, DCX, DDC, DDHD2, DDOST, DDX11, DDX3X, DEAF1, DEPDC5, DHCR24, DHCR7, DHFR, DHTKD1, DHX30, DIAPH1, DIS3L2, DKC1, DLD, DLG3, DMD, DNAJC12, DNAJC19, DNM1, DNMT3A, DNMT3B, DOCK7, DOCK8, DOLK, DPAGT1, DPM1, DPP6, DPYD, DYM, DYNC1H1, DYRK1A, EBF3, EBP, EDNRB, EED, EEF1A2, EFTUD2, EHMT1, EIF2AK3, EIF2S3, ELAC2, ELOVL4, ELP2, EML1, EMX2, EP300, EPG5, ERCC1, ERCC2, ERCC3, ERCC5, ERCC6, ERCC6L2, ERCC8, ESCO2, ETFA, ETFB, ETFDH, ETHE1, EXOSC3, EXTL3, EZH2, FAM111A, FAM126A, FAM20C, FAR1, FAT4, FBXL4, FGD1, FGF12, FH, FIG4, FKRP, FKTN, FLNA, FLVCR1, FLVCR2, FMN2, FMR1, FOLR1, FOXG1, FOXP1, FOXP2, FOXRED1, FTCD, FTSJ1, FUCA1, GABRA1, GABRB3, GABRG2, GALC, GALE, GALT, GAMT, GATAD2B, GATM, GCDH, GCH1, GDI1, GFAP, GFER, GFM1, GJC2, GK, GLB1, GLDC, GLI2, GLI3, GLIS3, GLUL, GLYCTK, GM2A, GMPPA, GMPPB, GNAO1, GNAS, GNB1, GNPAT, GNPTAB, GNPTG, GNS, GPAA1, GPC3, GPSM2, GRIA3, GRID2, GRIK2, GRIN1, GRIN2A, GRIN2B, GRM1, GSPT2, GTF2H5, GTPBP3, GUSB, HACE1, HADH, HADHA, HCCS, HCFC1, HCN1, HDAC4, HDAC8, HECW2, HERC1, HESX1, HEXA, HEXB, HGSNAT, HIBCH, HIST1H1E, HIVEP2, HLCS, HMGCL, HNRNPH2, HNRNPU, HOXA1, HPD, HPRT1, HRAS, HSD17B10, HSD17B4, HSPD1, HTRA2, HUWE1, HYLS1, IARS, IDH2, IDS, IDUA, IER3IP1, IFIH1, IFT172, IGF1, IKBKG, IL1RAPL1, INPP5E, INPP5K, IQSEC2, IRX5, ISPD, ITPR1, IVD, JAM3, KANSL1, KAT6A, KAT6B, KCNA2, KCNB1, KCNC1, KCNC3, KCNH1, KCNJ10, KCNJ11, KCNJ6, KCNK9, KCNQ2, KCNT1, KCTD7, KDM5B, KDM5C, KDM6A, KIAA0586, KIAA1109, KIAA2022, KIDINS220, KIF11, KIF1A, KIF1BP, KIF5C, KIF7, KMT2A, KMT2C, KMT2D, KNL1, KPTN, KRAS, L1CAM, L2HGDH, LAMA1, LAMA2, LAMB1, LAMC3, LAMP2, LARGE1, LARP7, LGI4, LIG4, LINS1, LONP1, LRP2, LRPPRC, MAB21L2, MAF, MAGEL2, MAN1B1,

Document S1: List of genes in the intellectual disability panel

MAN2B1, MANBA, MAOA, MAP2K1, MAP2K2, MASP1, MAT1A, MBD5, MBOAT7, MBTPS2, MCCC1, MCCC2, MCOLN1, MCPH1, MDH2, MECP2, MED12, MED13L, MED17, MEF2C, MFF, MFSD8, MGAT2, MGP, MICU1, MID1, MKKS, MKS1, MLC1, MLYCD, MMAA, MMAB, MMACHC, MMADHC, MOCS1, MOCS2, MOGS, MPDU1, MPI, MPLKIP, MRPS22, MSMO1, MTHFR, MTOR, MTR, MTRR, MUT, MVK, MYCN, MYO5A, MYT1L, NAA10, NACC1, NAGA, NAGLU, NALCN, NANS, NDE1, NDP, NDST1, NDUFA1, NDUFS1, NDUFS4, NDUFS7, NDUFS8, NDUFV1, NEU1, NF1, NFIA, NFIX, NFU1, NGLY1, NHS, NIPBL, NKX2-1, NLGN3, NONO, NPC1, NPC2, NPHP1, NR2F1, NRAS, NRXN1, NRXN2, NSD1, NSDHL, NSUN2, NT5C2, NTRK1, NUBPL, OCLN, OCRL, OFD1, OGT, OPA3, OPHN1, ORC1, ORC4, ORC6, OTC, OTUD6B, OTX2, PACS1, PAFAH1B1, PAH, PAK3, PARN, PAX6, PAX8, PC, PCCA, PCCB, PCDH19, PCGF2, PCNT, PDE4D, PDHA1, PDHX, PDSS1, PDSS2, PEPD, PEX1, PEX10, PEX11B, PEX12, PEX13, PEX14, PEX16, PEX19, PEX2, PEX26, PEX3, PEX5, PEX6, PEX7, PGAP1, PGAP2, PGAP3, PGK1, PHF6, PHF8, PHGDH, PIGA, PIGL, PIGN, PIGO, PIGT, PIGV, PIK3CA, PIK3R2, PLA2G6, PLAA, PLCB1, PLK4, PLP1, PMM2, PNKP, PNPT1, PNPLA6, POGZ, POLG, POLR3A, POLR3B, POMGNT1, POMGNT2, POMT1, POMT2, PORCN, PPM1D, PPP1CB, PPP2R1A, PPP2R5D, PPT1, PQBP1, PRKD1, PRMT7, PRODH, PROSC, PRPS1, PRRT2, PRSS12, PRUNE1, PSAP, PSMD12, PSPH, PTCH1, PTCHD1, PTDSS1, PTEN, PTF1A, PTPN11, PTS, PUF60, PURA, PUS1, PYCR1, PYCR2, QARS, QDPR, RAB11B, RAB18, RAB23, RAB39B, RAB3GAP1, RAB3GAP2, RAC1, RAD21, RAF1, RAI1, RANBP2, RARB, RARS2, RBM10, RELN, RERE, RFT1, RIT1, RLIM, RMND1, RNASEH2A, RNASEH2B, RNASEH2C, RNASET2, ROGDI, ROR2, RPGRIP1L, RPL10, RPS6KA3, RRM2B, RTEL1, RTTN, SAMD9, SAMHD1, SATB2, SC5D, SCN1A, SCN2A, SCN8A, SCO1, SCO2, SDHA, SDHAF1, SERAC1, SETBP1, SETD5, SGPL1, SGSH, SHANK1, SHANK2, SHANK3, SHH, SHOC2, SIK1, SIL1, SIN3A, SIX3, SKI, SLC12A5, SLC12A6, SLC13A5, SLC16A2, SLC17A5, SLC19A3, SLC22A5, SLC25A1, SLC25A15, SLC25A20, SLC25A22, SLC2A1, SLC33A1, SLC35A2, SLC35C1, SLC39A14, SLC39A8, SLC46A1, SLC4A4, SLC6A1, SLC6A17, SLC6A19, SLC6A3, SLC6A8, SLC6A9, SLC9A6, SLX4, SMAD4, SMARCA2, SMARCA4, SMARCB1, SMARCE1, SMC1A, SMC3, SMOC1, SMPD1, SMS, SNAP29, SNRPB, SNX14, SON, SOS1, SOX10, SOX11, SOX2, SOX3, SOX5, SOX9, SPG11, SPG20, SPR, SPRED1, SPTAN1, SPTBN2, SRCAP, SRD5A3, SRPX2, ST3GAL3, ST3GAL5, STAG1, STAMBP, STIL, STRA6, STX1B, STXBP1, SUCLG1, SUMF1, SUOX, SURF1, SYN1, SYNGAP1, SYNJ1, SYP, SZT2, TAF1, TANGO2, TAT, TAZ, TBC1D24, TBCD, TBCE, TBCK, TBL1XR1, TBR1, TCF4, TCN2, TCTN2, TECPR2, TGIF1, TH, THAP1, THOC2, THOC6, THRA, TIMM8A, TMCO1, TMEM165, TMEM216, TMEM237, TMEM240, TMEM5, TMEM67, TMEM70, TMTC3, TOE1, TPP1, TRAPPC9, TREX1, TRIM32, TRIM37, TRIO, TRIP12, TRIT1, TRMT10A, TSC1, TSC2, TSEN2, TSEN34, TSEN54, TSFM, TSHB, TSPAN7, TTC19, TTC37, TTC8, TTI2, TUBA1A, TUBA8, TUBB, TUBB2A, TUBB2B, TUBB3, TUBB4A, TUBGCP6, TUSC3, TWIST1, UBA5, UBE2A, UBE3A, UBE3B, UBR1, UBTF, UMPS, UNC80, UPF3B, UROC1, USP9X, VAMP1, VLDLR, VPS13B, VRK1, WAC, WDPCP, WDR45, WDR45B, WDR62, WDR73, WDR81, WWOX, XRCC4, YWHAG, YY1, ZBTB18, ZBTB20, ZC4H2, ZDHHC9, ZEB2, ZFYVE26, ZIC1, ZIC2, ZMYND11, ZNF711, ZSWIM6

Figure S3: Vcf2cytosure and array plots of illustrative cases. Screenshot from the Cytosure Interpret Software (Oxford Gene Technology, Oxfordshire, UK), with an array comparative genomic hybridization (array-CGH) plot shown on the left and a vcf2cytosure WGS representation to the right. Each dot represents an oligonucleotide probe (array-CGH) or a coverage bin (vcf2cytosure). Five illustrative CNVs identified in Cohort 3 are shown: a 3.45 Mb duplication on 2p21p22.1 (RD_P403), a 2.56 Mb duplication on 3p25.2p25.3 (RD_P404), a 1.44 Mb deletion on 7q11.23 (RD_P407), a 7.52 kb deletion on 8p11.22 (RD_P409) and a 2.65 Mb recurrent deletion on 22q11.21 (RD_P415). For case RD_P409 a 1M aCGH design was used and in the other four cases a 4x180 aCGH design.

RD_P403

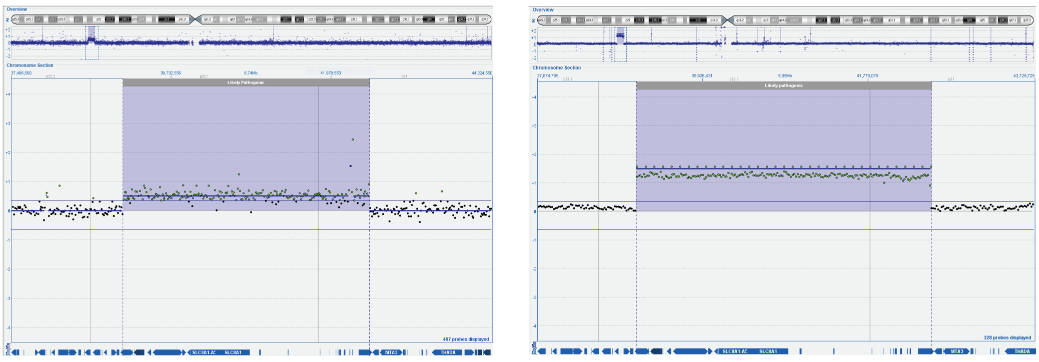

RD_P404

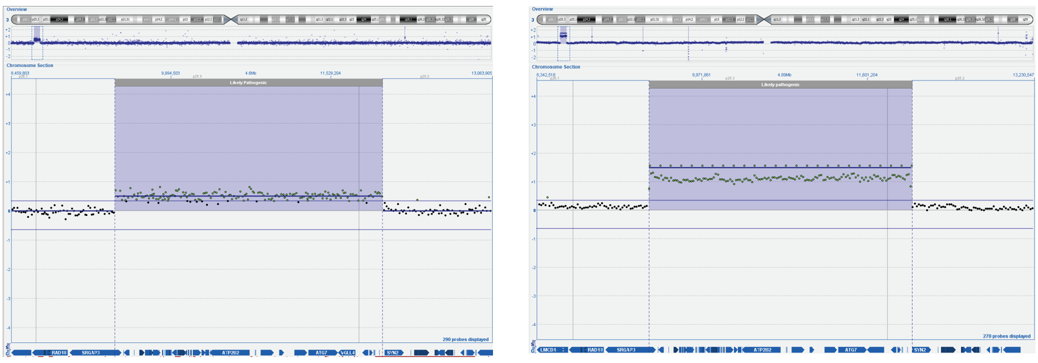

RD_P407

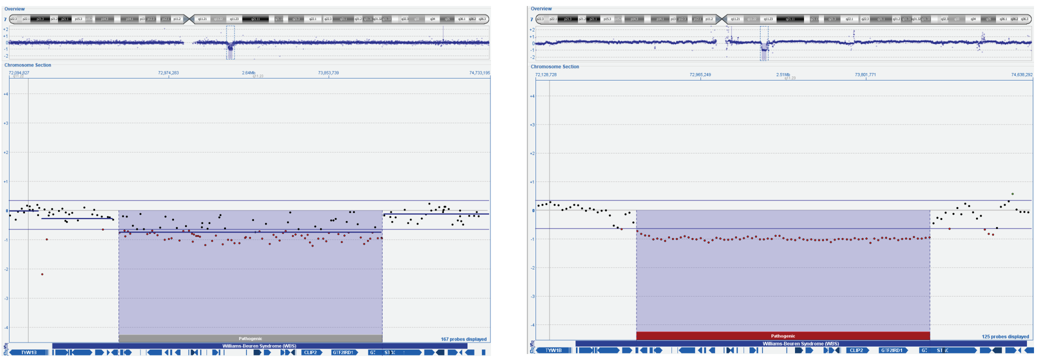

RD_P409

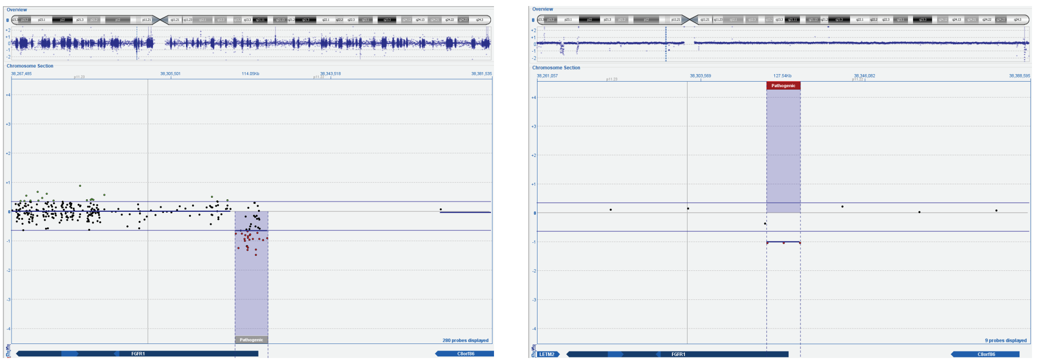

RD_P415

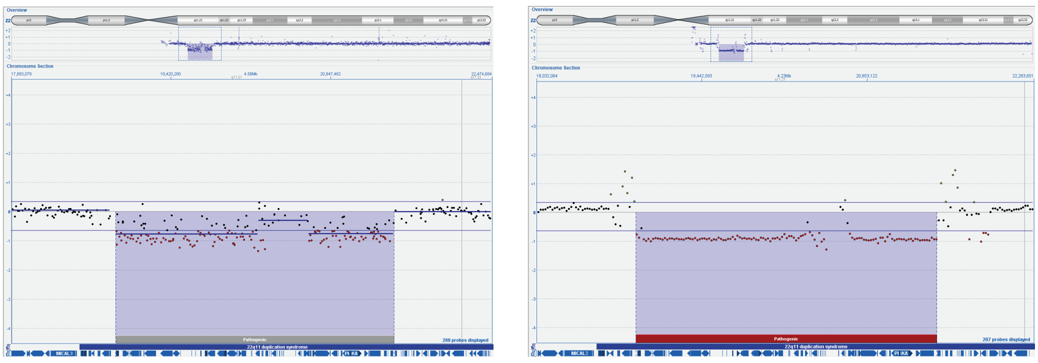

Figure S4: Breakpoint junction analysis of individuals RD_P77, RD_P405, RD_P414, RD_P406.
A. Sequence analysis of the 4-7 translocation in individual RD_P77. The two parental chromosomes are shown, chr4 (top) and chr 7 (bottom) and the derivative chromosome sequence in the middle. Insertions are present in both junctions of 7 bp and 5 bp, respectively (pink).
B. Sequence analysis of the 3q25.32q26.1 insertional duplication in individual RD_P405. Two junctions have blunt ends and a one nucleotide microhomology (lilac) is present in one junction.
C. Sequence analysis of the r(18) present in individual RD_P414. A one nucleotide insertion is seen in the junction.
D. Sequence analysis of the 4q25q35.2 unbalanced translocation in individual RD_P406. An insertion of four nucleotides (pink) is seen in the junction.

A RD_P77: deletion on chromosome 4 that is part of a cryptic t(4;7)

```
chr4:171307789_intergenic(+)   ACAGTCAGCTACGGGGAGATCGCTGCTTTAAGGCTATATTCATCAGCTTGGTGGTTAtgatgttttggtgatttatggatgtttatgactcacgtatggatacaaat
jct1                           ACAGTCAGCTACGGGGAGATCGCTGCTTTAAGGCTATATTCATCAGCTTGGTGGTTACCTTCCCAAAATATGCATATCATACTTAGGAAAATGTTATCCAAAGAGGT
chr7:9350275_L1(-)             ATTTCTAGTTATCAGCAATAAACAATCTGAAAATAAAATTTAGATAATTTCATTAACAATATATTAAATATGCATATCATACTTAGGAAAATGTTATCCAAAGAGGT

chr4:180641897_ERV1(+)         GACTCTGCCCACCAGAGAACCCCCATTTGACTGTAATTTTCCATTACCTTCCCAAATCTTATAAAACGGCCCCACCCCTATCTCCCTTTGCTGACTCTCTTTTCGGA
jct2                           AGCAATAAACAATCTGAAAATAAAATTTAGATAATTTCATTAACAAATTTACCAAATCTTATAAAACGGCCCCACCCCTATCTCCCTTTGCTGACTCTCTTTTCGGA
chr7:9350275_L1(-)             AGCAATAAACAATCTGAAAATAAAATTTAGATAATTTCATTAACAATATATTAAATATGCATATCATACTTAGGAAAATGTTATCCAAAGAGGTGCAAGCCTTCATA
```

B RD_P405: duplication on chromosome 3 inserted on chromosome 13 + inversion on chromosome 13

```
chr3:158562961_ERVL(+)          ATCTGATATGATTGGTGTTCTTATTAAAAGGAACACTGGCTGGGCACGGTGGCTCATGCCTGTAATCCCAGCTCTTTGGGAAGTAGAGGTGGGAGGATCATTTCAG
jct1                            ACAGGGGAAGGATGGAAGGAGAGAAAATAAAGAAGGGTAATCATTGTAGCGGCTCATGCCTGTAATCCCAGCTCTTTGGGAAGTAGAGGTGGGAGGATCATTTCAG
chr13:82234080_intergenic(+)    ACAGGGGAAGGATGGAAGGAGAGAAAATAAAGAAGGGTAATCATTGTAGCACCTCTTATATATATAGCAGAAAATTCAGAAGGCAAGTGTATAATACATGATAAAA

chr3:160802997_exon5_B3GALNT1(+) TAATTTTAAATAATAATAACTGTGAAATACTGCAACATCTTGAAGTACTTTATAAATGACCAAAAACAGGTAAAATTTTGTTCAGTATAACTTCAGTGAAGAAGTT
jct2                            TAATTTTAAATAATAATAACTGTGAAATACTGCAACATCTTGAAGTACTTTATAAATGCATTTTCAGTAAACTAACACAGGAACAGAAAACCAAACACTGCATGTT
chr13:82303653_L1(-)            TGGCCATAAAAAAGAATGGGTTCATGTCCATTTCAGGAACATGGATGAAGCTGGAAACCATTTTCAGTAAACTAACACAGGAACAGAAAACCAAACACTGCATGTT

chr13:82234087_intergenic(-)    GTATTATACACTTGCCTTCTGAATTTTCTGCTATATATATAAGAGGTGCTACAATGATTACCCTTCTTTATTTTCTCTCCTTCCATCCTTCCCCTGTTTCTCCTCC
jct3                            GTATTATACACTTGCCTTCTGAATTTTCTGCTATATATATAAGAGGTGTTTCCAGCTTCATCCATGTTCCTGAAATGGACATGAACCCATTCTTTTTTATGGCCAC
chr13:82303670_L1(+)            ACATGCAGTGTTTGGTTTTCTGTTCCTGTGTTAGTTTACTGAAAATGGTTTCCAGCTTCATCCATGTTCCTGAAATGGACATGAACCCATTCTTTTTTATGGCCAC
```

C RD_P414: ring chromosome with terminal 18p and 18q deletions

```
chr18:62969677_intergenic(+)  ACTTTGGGCCATTCTATGAAGCAAGTTTAACTATTTTTGGCTCTTAGTGACCACTAATATATTCACTCAATAAAGAAAGTTACTACTCCAGTAGTAATTCTGGCC
jct                           ACTTTGGGCCATTCTATGAAGCAAGTTTAACTATTTTTGGCTCTTAGTGACCACTGGGTCATTTTCTAGTCTTTTATTTTTTACACAGATACAAGGATTTATATT
chr18:1653327_intergenic(+)   ATTTGTTCCCTAGCATCATTTCTCTCACTACCTATGTACATTCAAATATGCCTACAGGTCATTTTCTAGTCTTTTATTTTTTACACAGATACAAGGATTTATATT
```

D RD_P406: duplication on chromosome 4 inserted on the chromosome 2p telomere

```
chr4:109934890_ERV1(-)  TGGTGAGTGTTACAGTTCTTAAAGATGGTGTGTCCGGAGTTTGTTCCTTCTGATGTTCGGACGTGCTTAGAGTTTCTTCCTTCGGTTGGGTTCGTGGTCTCGCTG
jct                     TGGTGAGTGTTACAGTTCTTAAAGATGGTGTGTCCGGAGTTTGTTGGCTGGGAAGAGTATTTGACTGCCAGTGTGGAATGAGGGGGACTTGGGATCACACTCTTC
chr2:26894_L1(+)        tcctttttcaatgtctgaagaaaataaattgtttctttttctgatgtcaGGGAAGAGTATTTGACTGCCAGTGTGGAATGAGGGGGACTTGGGATCACACTCTTC
```

Document S2

CLINICAL DESCRIPTIONS

Individual RD_P77 with a deletion on chromosome 4 and a cryptic t(4;7)(q33p21.3)

The patient was born as the 2nd child to healthy non-consanguineous parents of Kurd origin. He was born at full term after an uneventful pregnancy and his birth weight was 4050 g (+1,37 z-score) and birth length 51 cm (+0,59 z-score). Atresia of pulmonary artery with intact ventricular septum was diagnosed prenatally through fetal echocardiography and he underwent his first surgical procedure directly after birth. There were severe feeding difficulties during early childhood and he was fed through a nasogastric tube. He was operated on due to his pulmonary atresia three times. Clinical examination of the hands showed proximally placed thumbs caused by brachymetacarpia of dig 1 and symphalangism with stiffness and hypoplasia of hypothenar muscles and dig V bilaterally. Radiographs of the upper extremities were othervise normal. He also had abnormalities of his feet with overlapping dig II and III, and proximal partial syndactyly between dig II and II. Radiographs of the feet showed that the middle phalanx of dig V was missing bilaterally. From 5 months of age, he showed signs of impaired psychomotor development. He was able to sit without support only for short moments at 13 months. His speech development was delayed and he did not say any words for the first 2.5 years. At age 6 he has started to talk with long sentences. He has a short attention span but his cognitive level has not been formally assessed. He had one episode of febrile seizure during gastroenteritis but no other clinical manifestations of epilepsy. MRI of the brain was normal. He was operated due to retentio testis on his right side and he had surgical repair of a unilateral inguinal hernia. He has patches of alopecia on his head. He has asthma since early childhood that is treated with inhaled corticosteroids. He is also treated with laxatives to prevent problems with constipation. Auditory examination showed normal hearing. Ultrasound imaging of the abdomen was normal. Radiography of his neck was performed since his neck is stiff but showed normal results. Array comparative genomic hybridization (aCGH) was performed and showed a de novo 9,32 Mb interstitial deletion of chromosome 4 (4q33-q34.3).

Individual RD_P393 with a DEL-INV-DEL on 2q24

The second child to nonrelated parents. She was born term after normal pregnancy. She was of normal length, weight and head circumference (6d of age, weight 3840g (+1,03 z-score, length 53,2cm (+1,61 z-score) head circumference 34,5cm (+0,04 z-score)). Delivery was uncomplicated and she left the hospital after a few hours. She had her first seizure the first day of life. The seizure included an absence, stiffness and a long apnea, that did not respond to stimulation. Initial newborn screening was normal. She had severe severe muscular hypotonia from birth. MRI performed at 3 months showed normal morphology. Multiple EEGs did not reveal any consistent epileptic, or seizure activities during the first year of life. Muscle biopsy and analysis of mtDNA did not reveal any mitochondrial defect. Metabolic screening was normal. Whole genome sequencing filtered for inborn errors of metabolism did not indicate any metabolic disease. The seizures later on include a combination of apneas, absenses, focal seizures, myoclonias and general seizures. The seizures did not respond to anti-epileptic medications including pyridoxin, folic acid, fenobarbital, levetiracetam, oxcarbazepine, clonazepam, clobazam, topiramate, valproate, zonisamide, or stiripentol. From 6 months of age the growth of the head slowed down (at one year 11 months 45.9cm (-

0,78, z-score). Delayed development was noticed. A follow up MRI at 9 months of age showed delayed myelinization and wide lateral and side ventricles. At the same time EEG revealed a slow background, with interictal multifocal epileptic activity and ictal polyspike and waves upon photostimulation. She is currently 3 years old and has very little voluntary movements, no speach and a severe developmental delay.

Individual RD_P400 with a heterozygous deletion on chromosome 2 including *NPPC*

The patient was the second of three children to nonconsanguineous parents of Swedish origin. She was born from uncomplicated pregnancy at GA 42 weeks, birth weight 3,75 kg (z-score +0,34), birth length 50 cm (-0,79 z-score), and head circumference 33 cm (-1,94 z-score). The delivery was uncomplicated, but after birth she needed short CPAP treatment due to respiratory problems. She started to walk independently at 14 month of age. Up to 2 yo she had frequent otitis media and needed ear tubes, and she suffers from atopic eczema which she has inherited from her father. Since 2 yo she had difficulties to sleep, and was treated with melatonin. She was referred to endocrine evaluation at age 7y8 months because of mild disproportionate short stature, with small hands and feet and the basic endocrine evaluation was normal. Additional symptoms included sleeping difficulties, learning difficulties, and mildly delayed developmental milestones. Whole genome sequencing revealed a 2q34 deletion of 282kb, inherited from her mother, whose adult height was 163,9 cm (+1,2 z-score), and who also had small hands and feet, but was otherwise healthy. The patients maternal grandmothers height was 153 cm (-0,55 z-score). At 7,5 yo she was 113,4 (-0,82 z-score (-2,5 z-score for Swedish growth standard (1)) cm in height, 24,6 kg (z-score +0,22) in weight, her sitting height was 64.2 cm, and relative sitting height 56.6% (corresponding to +2,5 SD (2)). Her skeletal survey showed only mild abnormalities such as slightly delayed maturation of vertebral bodies on lateral spine radiogram and mild brachydactyly, which was clinically considered as idiopathic short stature. The proband is currently 10 y and 9 month old and has also been treated because of strabismus and has hyperopic astigmatism.

Individual RD_P431 with an STR in ATXN7

The female patient is third child to unrelated parents originating from Bangladesh. She was born term after a pregnancy complicated by preeclampsia and high maternal glucose levels. Birth weight was 3180g (-0.1 z-score) and birth length was 51cm (-0,99 z-score). Newborn screening was normal. Early development was mostly normal with eye contact and the ability to turn from stomach to back at 3-4 months of age. Then a regression in these abilities was noted and she her muscular hypotonia increased. At 5 months she was referred for further investigation due to motor developmental delay, muscle hypotonia and nystagmus. At 7 months of age she was found to have a systolic cardiac murmur and ECG indicated left ventricular hypertrophy. Cardiac ultrasound revealed a large persistent ductus and a secondary atrial septum defect. The blood tests showed increased white cell count and metabolic acidosis. Metabolic investigation and muscle biopsy showed findings consistent with a defect in the respiratory chain. She deceased at 10months of age due to cardiac insufficiency and arrest.

Document S2

The father of RD_P431 is a 41 years old man originally from Bangladesh with loss of vision starting at age 36. Ophthalmological examination revealed bilateral decreased visual acuity and poor color vision. ERG confirms the presence of cone-rod dystrophy. Genetic tests for Leber's hereditary optic neuropathy and *OPA1* were negative.

Web resources

WHO auxology tables were used for the calculations of z-score:
https://www.who.int/childgrowth

References

1. Wikland KA, Luo ZC, Niklasson A, Karlberg J. Swedish population-based longitudinal reference values from birth to 18 years of age for height, weight and head circumference. Acta Paediatr. 2002;91(7):739-54.
2. Fredriks AM, van Buuren S, van Heel WJ, Dijkman-Neerincx RH, Verloove-Vanhorick SP, Wit JM. Nationwide age references for sitting height, leg length, and sitting height/height ratio, and their diagnostic value for disproportionate growth disorders. Arch Dis Child. 2005;90(8):807-12.
